# A New Series of Strigolactone Analogs Derived From Cinnamic Acids as Germination Inducers for Root Parasitic Plants

**DOI:** 10.3389/fpls.2022.843362

**Published:** 2022-03-29

**Authors:** Taiki Suzuki, Michio Kuruma, Yoshiya Seto

**Affiliations:** Laboratory of Plant Chemical Regulation, School of Agriculture, Meiji University, Kawasaki, Japan

**Keywords:** strigolactone, *cis*-cinnamic acid, root parasitic plant, germination, *Orobanche*, *Striga*

## Abstract

Root parasitic plants such as *Striga* and *Orobanche* cause significant damage on crop production, particularly in sub-Saharan Africa. Their seeds germinate by sensing host root-derived signaling molecules called strigolactones (SLs). SL mimics can be used as suicidal germination inducers for root parasitic plants. Previous attempts to develop such chemicals have revealed that the methylbutenolide ring (D-ring), a common substructure in all the naturally occurring SLs, is critical for SL agonistic activity, suggesting that it should be possible to generate new SL mimics simply by coupling a D-ring with another molecule. Because structural information regarding SLs and their receptor interaction is still limited, such an approach might be an effective strategy to develop new potent SL agonists. Here, we report development of a series of new SL analogs derived from cinnamic acid (CA), the basis of a class of phenylpropanoid natural products that occur widely in plants. CA has an aromatic ring and a double-bond side-chain structure, which are advantageous for preparing structurally diverse derivatives. We prepared SL analogs from *cis* and *trans* configuration CA, and found that all the *cis*-CA-derived SL analogs had stronger activities as seed germination inducers for the root parasitic plants, *Orobanche minor* and *Striga hermonthica*, compared with the corresponding *trans*-CA-derived analogs. Moreover, introduction of a substitution at the C-4 position increased the germination-stimulating activity. We also found that the SL analogs derived from *cis*-CA were able to interact directly with SL receptor proteins more effectively than the analogs derived from *trans*-CA. The *cis* isomer of CA was previously reported to have a growth promoting effect on non-parasitic plants such as *Arabidopsis*. We found that SL analogs derived from *cis*-CA also showed growth promoting activity toward *Arabidopsis*, suggesting that these new SL agonists might be useful not only as suicidal germination inducers for root parasitic weeds, but also as plant growth promoters for the host plants.

## Introduction

Root parasitic plants such as *Striga* and *Orobanche* parasitize the root of their host plants, which include some important crops such as rice, sorghum, and maize. After invasion into the host, parasitic plants connect their xylem tissue to the vascular tissue of the host plant, and obtain water and nutrients from the host plant *via* this xylem bridge. They produce numerous tiny seeds, which are spread onto the field. The seeds can stay dormant for decades, but once the host plant is planted nearby, they germinate by sensing strigolactone (SL) molecules that are released from the host root (Cook et al., 1966). If the germinated seeds cannot attach to the host, they die within 4–5 days. On the basis of this germination process, suicidal germination induction has been proposed as an effective way to eliminate parasitic plant seeds from infested fields. However, because quantitative production of the SL molecules has become a bottleneck, this method has not been put into practical use.

After the initial discovery of SLs as germination inducers for root parasitic plants, SLs were further shown to be the symbiotic signals for arbuscular mycorrhizal fungi (Akiyama et al., 2005). Moreover, SLs were identified as a new class of plant hormones that regulate shoot branching (Gomez-Roldan et al., 2008; Umehara et al., 2008). After the discovery of SLs as plant hormones, progress in this research field has included uncovering the SL signaling mechanism mediated by an α/β-hydrolase type receptor. In non-parasitic plants, DWARF14 (D14), a member of this protein family, was identified as the SL receptor (Arite et al., 2009; Hamiaux et al., 2012; de Saint Germain et al., 2016; Yao et al., 2016; Seto et al., 2019; Mashiguchi et al., 2021). In addition, a paralogous family of D14, which is called HYPOSENSITIVE TO LIGHT/KARRIKIN INSENSITIVE2 (hereafter denoted HTL), was characterized as a receptor for the smoke-derived germination inducer, karrikin (KAR; Waters et al., 2012). In a root parasitic plant, *Striga hermonthica*, 11 *HTL* genes were found, some of which were characterized as receptors for SL molecules, but KAR was not (Conn et al., 2015; Toh et al., 2015; Tsuchiya et al., 2015). Moreover, one of those HTLs, ShHTL7, was identified as an extremely sensitive receptor for SLs with pM sensitivity when expressed in *Arabidopsis* (Toh et al., 2015).

**Figure 1:**
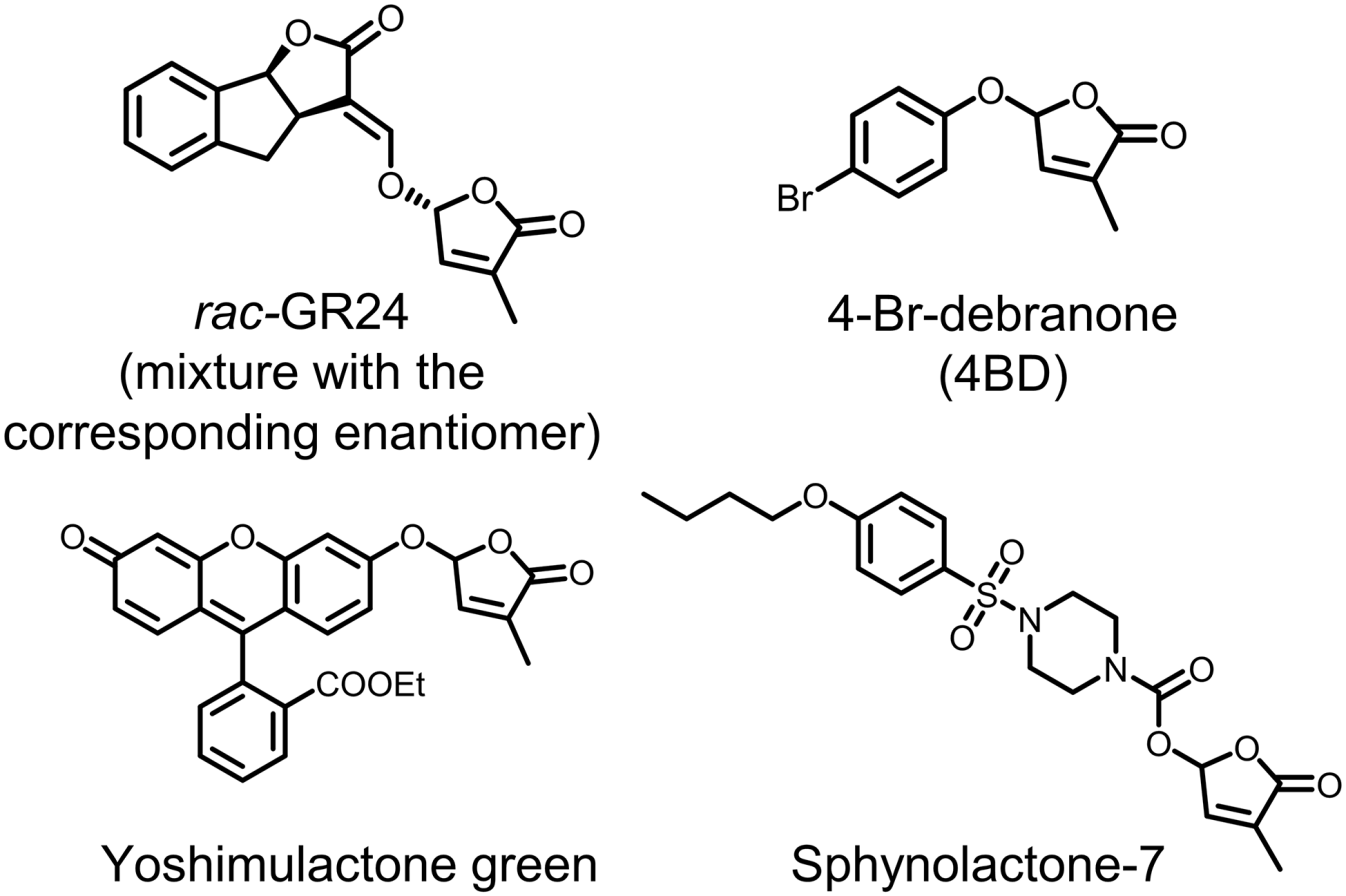
Chemical structures of the synthetic strigolactone (SL) analogs.

Many synthetic SL agonists have been reported, among which GR24 is now commonly used in basic research as a positive control (Figure 1). Such attempts have revealed that the methylbutenolide (D-ring) part, a common component in all the reported naturally occurring SLs, has a critical role for SL agonistic activity. Although the enol ether bridge connecting the ABC tricyclic ring, a typical part in the canonical SL molecules, and the D-ring has been proposed to be an essential structural feature for SL analogs, debranones, in which the D-ring is connected to a phenolic group *via* a simple ether bridge, have been also reported as SL agonists (Figure 1; Fukui et al., 2011). Moreover, yoshimulactone green (YLG), in which the ABC-ring part is replaced with fluorescein, was reported to be a pro-fluorescence SL agonist (Figure 1). YLG retains SL agonistic activity both for shoot branching inhibition and for inducing germination in parasitic plants, and it can be hydrolytically cleaved by the D14 and the *Striga* HTL receptors (Tsuchiya et al., 2015). YLG hydrolysis emits fluorescence and it enables high-throughput detection of the receptor function. The discovery of the above-mentioned *Striga* receptors was accomplished using this unique analog (Tsuchiya et al., 2015). Moreover, the receptor identification, combined with YLG, enabled a rapid screening of chemicals that can directly interact with HTL7. By this approach, sphynolactone-7 (SPL7) was discovered as an extremely strong SL agonist that can induce *S. hermonthica* germination at fM concentrations (Uraguchi et al., 2018).

Because the detailed mechanism of receptor-ligand interactions in SL perception has not yet been uncovered, an effective strategy to obtain new potential agonist molecules would be to screen chemicals containing the D-ring structure. Using this approach, here we report development of a new type of SL analog, in which a cinnamic acid (CA) moiety is simply coupled with the D-ring through the carboxylic acid part *via* an ester bond. CA is widely distributed in plant species, and is a precursor of many important plant molecules such as lignin, flavonoids, isoflavonoids, and coumarins. CA exists as both *trans* and *cis* isomers, of which the former is more stable and common in nature. This structural feature enabled us to prepare two types of conformationally distinct analogs. In addition, many different analogs with substitutions on the benzene ring are commercially available. Thus, we were able to prepare a variety of structurally diverse analogs with a common core skeleton. A further feature of interest was that the *cis* isomer of CA was recently reported to act as an auxin efflux inhibitor, which leads to growth-promoting activity at low concentration (Steenackers et al., 2017, 2019). However, at a high concentration, *cis*-CA showed growth-inhibiting activity, and *cis*-CA was identified as an allelopathic compound that suppresses the growth of neighboring plants (Hiradate et al., 2005). Building on these observations, we chose CA as the starting material for synthesizing new SL analogs. These new synthetic analogs showed germination-inducing activity towards two root parasitic plants, *Orobanche minor* and *Striga hermonthica*, at moderately low concentration. Moreover, the *cis*-CA-derived SL analog showed a growth promoting effect on *Arabidopsis*, possibly as a result of degradation to *cis*-CA in the growth medium and in planta. Thus, we expect that these new SL agonists might provide lead chemicals for developing a new type of suicidal germination inducers with an additional function as growth promoters in the host plant.

## Materials and Methods

### Preparation of *cis*-Isomers of CA

*cis*-isomers of CA derivatives were individually prepared by isomerization from the corresponding *trans*-isomer. Each *trans*-CA (300–500 mg) was dissolved in MeOH or CH_3_CN (50 ml). The solution was placed under UV lamp (254 nm). After the irradiation for 16 h, the solvent was evaporated *in vacuo*, and the mixture of *trans*/*cis* CA was suspended with 5–10 ml of distilled water. The sample was sonicated for 5 min and then centrifuged with 18,000 *g* for 30 min. The supernatant was filtered and diluted with distilled water up to 30 ml. The pH was adjusted to 1 using 1 N HCl and the sample was extracted with ethyl acetate (3 × 30 ml). The organic layer was dried over Na_2_SO_4_ and concentrated *in vacuo*, and the obtained each *cis*-CA was further subjected to the D-ring coupling reaction.

### Chemical Synthesis of CASLs

5-Bromo-3-methyl-2(5H)-franone; 3-methyl-2(5H)-furanone (5 g, 51 mmol) was added to the solution of *N*-bromosuccinimide (9.05 g, 51 mmol) and azobis(isobutyronitrile) (170 mg, 1.02 mmol) in CCl_4_ (51 ml). The mixture was refluxed at 90°C for 4 h. After the reaction, the mixture was cooled to room temperature and filtered. The filtrate was concentrated *in vacuo* and purified by silica gel column chromatography (*n*-hexane/EtOAc:8/2) to afford 5-bromo-3-methyl-2(5H)-franone (8.6 g, 95%). ^1^H-NMR (300 MHz, CDCl_3_) 𝛿 7.24 (t, *J* = 1.8, 1 H), 6.85 (q, *J* = 1.5 Hz, 1 H), 2.07 (m, 3 H).

*t*-CASL (*t*-CASL1); K_2_CO_3_ (1.0 mmol) was added to the solution of *trans*-CA (0.50 mmol) in *N*-methyl-2-pyrrolidone (5 ml). 5-Bromo-3-methyl-2(5H)-franone (0.98 mmol) diluted in *N*-methyl-2-pyrrolidone (5 ml) was added to the mixture, and the mixture was stirred for 12 h at room temperature. The reaction was quenched by adding 1 N HCl, and the solution was diluted to 50 ml with water. The sample was extracted with EtOAc (3 × 50 ml). The organic layer was washed with distilled water (3 × 150 ml), dried over Na_2_SO_4_, and concentrated *in vacuo*. The crude sample was purified by silica gel column chromatography (*n*-hexane/EtOAc:8/2) to afford *t*-CASL (99.8 mg, 0.41 mmol, 82%). ^1^H-NMR (300 MHz, CDCl_3_) 𝛿 7.79 (d, *J* = 16 Hz, 1 H), 7.56–7.52 (m, 2 H), 7.43–7.39 (m, 2 H), 7.03 (t, *J* = 1.5 Hz, 1 H), 6.99 (t, *J* = 1.5 Hz, 1 H), 6.43 (d, *J* = 16 Hz, 1 H), 2.02 (t, *J* = 1.5 Hz, 3 H); ^13^C-NMR (75 MHz, CDCl_3_), 𝛿 10.54, 92.52, 115.82, 128.26, 128.91, 130.92, 133.64, 134.27, 142.19, 147.42, 164.76, 171.12; HRMS [ESI+ (*m*/*z*)] calculated for (C_14_H_12_O_4_ + H)^+^ 245.0808, found 245.0818.

*c*-CASL (*c*-CASL1); K_2_CO_3_ (1.34 mmol) was added to the solution of *cis*-CA (0.67 mmol) in *N*-methyl-2-pyrrolidone (6.7 ml). 5-Bromo-3-methyl-2(5H)-franone (1.31 mmol) diluted in *N*-methyl-2-pyrrolidone (6.7 ml) was added to the mixture, and the mixture was stirred for 12 h at room temperature. The reaction was quenched by adding 1 N HCl, and the solution was diluted to 50 ml with water, extracted with EtOAc (3 × 50 ml). The organic layer was washed with distilled water (3 × 150 ml) and dried over Na_2_SO_4_ and concentrated *in vacuo*. The crude sample was purified by silica gel column chromatography (*n*-hexane/EtOAc:8/2) to afford *c*-CASL (96.0 mg, 0.39 mmol, 59%). ^1^H-NMR (300 MHz, CDCl_3_) 𝛿 7.64–7.61 (m, 2 H), 7.38–7.36 (m, 3 H), 7.12 (d, *J* = 13 Hz, 1 H), 6.92 (t, *J* = 1.2 Hz, 1 H), 6.86 (t, *J* = 1.8 Hz, 1 H), 5.94 (d, *J* = 13 Hz, 1 H), 1.97 (t, *J* = 1.2 Hz, 3 H); ^13^C-NMR (75 MHz, CDCl_3_), 𝛿 10.55, 92.26, 117.11, 128.00, 128.92, 129.63, 129.96, 134.16, 142.18, 146.99, 163.68, 171.09; HRMS [ESI+ (*m*/*z*)] calculated for (C_14_H_12_O_4_ + H)^+^ 245.0808, found 245.0815.

PPASL; K_2_CO_3_ (1.0 mmol) was added to the solution of PPA (0.5 mmol) in *N*-methyl-2-pyrrolidone (5 ml). 5-Bromo-3-methyl-2(5H)-franone (0.98 mmol) diluted in *N*-methyl-2-pyrrolidone (5 ml) was added to the mixture, and the mixture was stirred for 12 h at room temperature. The reaction was quenched by adding 1 N HCl, and the solution was diluted to 50 ml with water, extracted with EtOAc (3 × 50 ml). The organic layer was washed with distilled water (3 × 150 ml) and dried over Na_2_SO_4_ and concentrated *in vacuo*. The crude sample was purified by silica gel column chromatography (*n*-hexane/EtOAc:7/3) to afford PPASL (113.0 mg, 0.46 mmol, 92%). ^1^H-NMR (300 MHz, CDCl_3_) 𝛿 7.33–7.18 (m, 5 H), 6.87–6.83 (m, 2 H), 2.98 (t, *J* = 1.8 Hz, 2 H), 2.74–2.68 (m, 2 H), 1.98 (t, *J* = 1.5 Hz, 3 H); ^13^C-NMR (75 MHz, CDCl_3_), 𝛿 10.50, 30.36, 35.41, 92.24, 126.38, 128.18, 128.48, 134.21, 139.67, 141.95, 170.96, 170.99; HRMS [ESI+ (*m*/*z*)] calculated for (C_14_H_14_O_4_ + H)^+^ 247.0965, found 247.0965.

*trans*-Me-CASL (*t*-CASL2); K_2_CO_3_ (1.0 mmol) was added to the solution of *trans*-Me-CA (0.5 mmol) in *N*-methyl-2-pyrrolidone (5 ml). 5-Bromo-3-methyl-2(5H)-franone (0.98 mmol) diluted in *N*-methyl-2-pyrrolidone (5 ml) was added to the mixture, and the mixture was stirred for 12 h at room temperature. The reaction was quenched by adding 1 N HCl, and the solution was diluted to 50 ml with water, extracted with EtOAc (3 × 50 ml). The organic layer was washed with distilled water (3 × 150 ml) and dried over Na_2_SO_4_ and concentrated *in vacuo*. The crude sample was purified by silica gel column chromatography (*n*-hexane/EtOAc:7/3) to afford *trans*-Me-CASL (50.8 mg, 0.20 mmol, 39%). ^1^H-NMR (300 MHz, CDCl_3_) 𝛿 7.76 (d, *J* = 17 Hz, 1 H), 7.43 (d, *J* = 8.1 Hz, 2 H), 7.20 (d, *J* = 8.1 Hz, 2 H), 7.02 (t, *J* = 1.5 Hz, 1 H), 6.98 (t, *J* = 1.5 Hz, 1 H), 6.37 (d, *J* = 16 Hz, 1 H), 2.38 (s, 3 H), 2.01 (t, *J* = 1.5 Hz, 3 H); ^13^C-NMR (75 MHz, CDCl_3_), 𝛿 10.51, 21.39, 92.47, 114.64, 128.12, 128.26, 128.31, 129.62, 130.94, 134.18, 141.50, 142.23, 147.42, 164.90, 171.11; HRMS [ESI+ (*m*/*z*)] calculated for (C_15_H_14_O_4_ + H)^+^ 259.0965, found 259.0978.

*cis*-Me-CASL (*c*-CASL2); K_2_CO_3_ (1.2 mmol) was added to the solution of *cis*-Me-CA (0.6 mmol) in *N*-methyl-2-pyrrolidone (6 ml). 5-Bromo-3-methyl-2(5H)-franone (1.18 mmol) diluted in *N*-methyl-2-pyrrolidone (6 ml) was added to the mixture, and the mixture was stirred for 12 h at room temperature. The reaction was quenched by adding 1 N HCl, and the solution was diluted to 50 ml with water, extracted with EtOAc (3 × 50 ml). The organic layer was washed with distilled water (3 × 150 ml) and dried over Na_2_SO_4_ and concentrated *in vacuo*. The crude sample was purified by silica gel column chromatography (*n*-hexane/EtOAc:8/2) to afford *cis*-Me-CASL (70.4 mg, 0.27 mmol, 45%). ^1^H-NMR (300 MHz, CDCl_3_) 𝛿 7.58 (d, *J* = 8.1 Hz, 2 H), 7.18 (d, *J* = 8.1 Hz, 7.08–7.04 (m, 1H), 6.93 (t, *J* = 1.5 Hz, 1 H), 6.89 (t, *J* = 1.5 Hz, 1 H), 5.87 (d, *J* = 12 Hz, 1 H), 2.37 (d, 3.9 Hz, 3 H), 1.97 (t, *J* = 1.5 Hz, 3 H); ^13^C-NMR (75 MHz, CDCl_3_), 𝛿 10.52, 21.37, 92.25, 115.92, 128.76, 128.79, 130.29, 130.34, 131.20, 134.15, 140.20, 142.11, 147.28, 163.79, 171.12; HRMS [ESI+ (*m*/*z*)] calculated for (C_15_H_14_O_4_ + H)^+^ 259.0965, found 259.0976.

*trans*-OH-CASL (*t*-CASL3); K_2_CO_3_ (1.0 mmol) was added to the solution of *trans*-OH-CA (0.5 mmol) in *N*-methyl-2-pyrrolidone (5 ml). 5-Bromo-3-methyl-2(5H)-franone (0.98 mmol) diluted in *N*-methyl-2-pyrrolidone (5 ml) was added to the mixture, and the mixture was stirred for 12 h at room temperature. The reaction was quenched by adding 1 N HCl, and the solution was diluted to 50 ml with water, extracted with EtOAc (3 × 50 ml). The organic layer was washed with distilled water (3 × 150 ml) and dried over Na_2_SO_4_ and concentrated *in vacuo*. The crude sample was purified by a silica gel column chromatography (*n*-hexane/EtOAc:6/4) to afford *trans*-OH-CASL (41.8 mg, 0.16 mmol, 32%). ^1^H-NMR (300 MHz, CDCl_3_) 𝛿 7.72 (d, *J* = 16 Hz, 1 H), 7.43 (d, *J* = 8.4 Hz, 2 H), 7.03–6.99 (m, 2 H), 6.87 (d, *J* = 8.7 Hz, 2 H), 6.26 (d, *J* = 16 Hz, 1 H), 6.16 (brs, 1 H), 2.02 (d, *J* = 1.2 Hz, 3 H); ^13^C-NMR (75 MHz, CDCl_3_), 𝛿 10.49, 93.34, 113.41, 116.75, 126.50, 131.42, 134.28, 144.22, 147.97, 161.13, 165.72, 171.91; HRMS [ESI+ (*m*/*z*)] calculated for (C_14_H_12_O_5_ + H)^+^ 261.0757, found 261.0771.

*cis*-OH-CASL (*c*-CASL3); K_2_CO_3_ (0.89 mmol) was added to the solution of *cis*-OH-CA (0.44 mmol) in *N*-methyl-2-pyrrolidone (4.5 ml). 5-Bromo-3-methyl-2(5H)-franone (0.87 mmol) diluted in *N*-methyl-2-pyrrolidone (4.5 ml) was added to the mixture, and the mixture was stirred for 3.5 h at room temperature. The reaction was quenched by adding 1 N HCl, and the solution was diluted to 50 ml with water, extracted with EtOAc (3 × 50 ml). The organic layer was washed with distilled water (3 × 150 ml) and dried over Na_2_SO_4_ and concentrated *in vacuo*. The crude sample was purified by silica gel column chromatography (*n*-hexane/EtOAc:6/4) and reverse phase HPLC (ODS SP-100, CH_3_CN/H_2_O:4/6). The HPLC purification was conducted for a part of the sample to afford *c*-CASL3 (5.2 mg, 0.02 mmol, 5%). ^1^H-NMR (300 MHz, acetone-D_6_) 𝛿 9.02 (s, 1 H), 7.82 (d, *J* = 9 Hz, 2 H), 7.23 (s, 1 H), 7.07 (d, *J* = 13 Hz, 1 H), 7.00 (s, 1 H), 6.87 (d, *J* = 8.4 Hz, 2 H), 5.81 (d, *J* = 13 Hz, 1 H), 1.94 (s, 3 H); ^13^C-NMR (75 MHz, acetone-D_6_), 𝛿 10.48, 93.28, 114.10, 115.81, 126.85, 134.17, 134.28, 144.14, 147.76, 160.29, 164.92, 171.90; HRMS [ESI+ (*m*/*z*)] calculated for (C_14_H_12_O_5_ + H)^+^ 261.0757, found 261.0772.

*trans*-OMe-CASL (*t*-CASL4); K_2_CO_3_ (1.0 mmol) was added to the solution of *trans*-OMe-CA (0.5 mmol) in *N*-methyl-2-pyrrolidone (5 ml). 5-Bromo-3-methyl-2(5H)-franone (0.98 mmol) diluted in *N*-methyl-2-pyrrolidone (5 ml) was added to the mixture, and the mixture was stirred for 12 h at room temperature. The reaction was quenched by adding 1 N HCl, and the solution was diluted to 50 ml with water, extracted with EtOAc (3 × 50 ml). The organic layer was washed with distilled water (3 × 150 ml) and dried over Na_2_SO_4_ and concentrated *in vacuo*. The crude sample was purified by a silica gel column chromatography (*n*-hexane/EtOAc:7/3) to afford *trans*-OMe-CASL (83.9 mg, 0.31 mmol, 61%). ^1^H-NMR (300 MHz, CDCl_3_) 𝛿 7.73 (d, *J* = 16 Hz, 1 H), 7.49 (d, *J* = 8.7 Hz, 2 H), 7.02 (t, *J* = 1.5 Hz, 1 H), 6.98 (d, *J* = 1.5 Hz, 1 H), 6.92 (d, *J* = 9.0 Hz, 2 H), 6.28 (m, 1 H), 3.84 (s, 3 H), 2.02 (d, *J* = 1.5 Hz, 3 H); ^13^C-NMR (75 MHz, CDCl_3_), 𝛿 10.59, 55.36, 92.51, 113.15, 114.40, 126.46, 130.11, 134.24, 142.31, 147.18, 161.92, 165.11, 171.20; HRMS [ESI+ (*m*/*z*)] calculated for (C_15_H_14_O_5_ + H)^+^ 275.0914, found 275.0927.

*cis*-OMe-CASL (*c*-CASL4); K_2_CO_3_ (1.9 mmol) was added to the solution of *cis*-OMe-CA (0.94 mmol) in *N*-methyl-2-pyrrolidone (9.4 ml). 5-Bromo-3-methyl-2(5H)-franone (1.84 mmol) diluted in *N*-methyl-2-pyrrolidone (9.4 ml) was added to the mixture, and the mixture was stirred for 12 h at room temperature. The reaction was quenched by adding 1 N HCl, and the solution was diluted to 50 ml with water, extracted with EtOAc (3 × 50 ml). The organic layer was washed with distilled water (3 × 150 ml) and dried over Na_2_SO_4_ and concentrated *in vacuo*. The crude sample was purified by silica gel column chromatography (*n*-hexane/EtOAc:7/3) to afford *cis*-OMe-CASL (154.5 mg, 0.56 mmol, 60%). ^1^H-NMR (300 MHz, CDCl_3_) 𝛿 7.76 (d, *J* = 8.7 Hz, 2 H), 7.00 (d, *J* = 13 Hz, 1 H), 6.95–6.86 (m, 4 H), 5.80 (d, *J* = 13 Hz, 1 H), 3.84 (d, *J* = 6.0 Hz, 3 H), 1.98 (t, *J* = 1.2 Hz, 3 H); ^13^C-NMR (75 MHz, CDCl_3_), 𝛿 10.54, 55.25, 92.26, 113.44, 114.08, 126.63, 132.73, 134.15, 142.20, 147.21, 160.99, 163.97, 171.17; HRMS [ESI+ (*m*/*z*)] calculated for (C_15_H_14_O_5_ + H)^+^ 275.0914, found 275.0926.

*trans*-OEt-CASL (*t*-CASL5); K_2_CO_3_ (1.0 mmol) was added to the solution of *trans*-OEt-CA (0.5 mmol) in *N*-methyl-2-pyrrolidone (5 ml). 5-Bromo-3-methyl-2(5H)-franone (0.98 mmol) diluted in *N*-methyl-2-pyrrolidone (5 ml) was added to the mixture, and the mixture was stirred for 12 h at room temperature. The reaction was quenched by adding 1 N HCl, and the solution was diluted to 50 ml with water, extracted with EtOAc (3 × 50 ml). The organic layer was washed with distilled water (3 × 150 ml) and dried over Na_2_SO_4_ and concentrated *in vacuo*. The crude sample was purified by silica gel column chromatography (*n*-hexane/EtOAc:7/3) to afford *trans*-OEt-CASL (83.9 mg, 0.29 mmol, 58%). ^1^H-NMR (300 MHz, CDCl_3_) 𝛿 7.73 (d, *J* = 16 Hz, 1 H), 7.47 (d, *J* = 8.7 Hz, 2 H), 7.02 (d, J = 1.5 Hz, 1 H), 6.98 (t, J = 1.5 Hz, 1 H), 6.90 (d, J = 8.7 Hz, 2 H), 6.27 (m, 1 H), 4.07 (q, J = 6.9 Hz, 2 H), 2.01 (t, *J* = 1.4 Hz, 3 H), 1.45–1.41 (m, 3 H); ^13^C-NMR (75 MHz, CDCl_3_), 𝛿 10.60, 14.63, 63.63, 92.52, 112.98, 114.56, 126.28, 130.12, 134.24, 142.31, 147.28, 161.35, 165.15, 171.21; HRMS [ESI+ (*m*/*z*)] calculated for (C_16_H_16_O_5_ + H)^+^ 289.1071, found 289.1082.

*cis*-OEt-CASL (*c*-CASL5); K_2_CO_3_ (0.76 mmol) was added to the solution of *cis*-OEt-CA (0.38 mmol) in *N*-methyl-2-pyrrolidone (3.8 ml). 5-Bromo-3-methyl-2(5H)-franone (0.75 mmol) diluted in *N*-methyl-2-pyrrolidone (3.8 ml) was added to the mixture, and the mixture was stirred for 12 h at room temperature. The reaction was quenched by adding 1 N HCl, and the solution was diluted to 50 ml with water, extracted with EtOAc (3 × 50 ml). The organic layer was washed with distilled water (3 × 150 ml) and dried over Na_2_SO_4_ and concentrated *in vacuo*. The crude sample was purified by a silica gel column chromatography (*n*-hexane/EtOAc:7/3) to afford *cis*-OEt-CASL (74.5 mg, 0.26 mmol, 68%). ^1^H-NMR (300 MHz, CDCl_3_) 𝛿 7.77–7.74 (m, 2 H), 7.08–6.87 (m, 5 H), 5.78 (d, *J* = 13 Hz, 1 H), 4.07 (q, *J* = 6.9 Hz, 2 H), 1.99 (t, *J* = 1.4 Hz, 3 H), 1.45–1.41 (m, 3 H); ^13^C-NMR (75 MHz, CDCl_3_), 𝛿 10.59, 14.67, 63.51, 92.29, 113.91, 113.97, 126.50, 132.83, 134.22, 142.21, 147.34, 160.47, 164.04, 171.20; HRMS [ESI+ (*m*/*z*)] calculated for (C_16_H_16_O_5_ + H)^+^ 289.1071, found 289.1083.

*trans*-NO_2_-CASL (*t*-CASL6); K_2_CO_3_ (1.0 mmol) was added to the solution of *trans*-NO_2_-CA (0.5 mmol) in *N*-methyl-2-pyrrolidone (5 ml). 5-Bromo-3-methyl-2(5H)-franone (0.98 mmol) diluted in *N*-methyl-2-pyrrolidone (5 ml) was added to the mixture, and the mixture was stirred for 12 h at room temperature. The reaction was quenched by adding 1 N HCl, and the solution was diluted to 50 ml with water, extracted with EtOAc (3 × 50 ml). The organic layer was washed with distilled water (3 × 150 ml) and dried over Na_2_SO_4_ and concentrated *in vacuo*. The crude sample was purified by silica gel column chromatography (*n*-hexane/EtOAc:7/3) to afford *trans*-NO_2_-CASL (72.6 mg, 0.25 mmol, 50%). ^1^H-NMR (300 MHz, CDCl_3_) 𝛿 8.27 (d, *J* = 8.7 Hz, 2 H), 7.81 (d, *J* = 16 Hz, 1 H), 7.70 (d, J = 8.7 Hz, 2 H), 7.03 (t, J = 1.5 Hz, 1 H), 6.99 (t, J = 1.5 Hz, 1 H), 6.55 (d, J = 16 Hz, 1 H), 2.03 (t, *J* = 1.2 Hz, 3 H); ^13^C-NMR (75 MHz, CDCl_3_), 𝛿 10.73, 92.79, 120.32, 124.30, 128.97, 134.75, 139.74, 141.82, 144.39, 148.89, 164.04, 170.97; HRMS [ESI+ (*m*/*z*)] calculated for (C_14_H_11_NO_6_ + H)^+^ 290.0659, found 290.0671.

*cis*-NO_2_-CASL (*c*-CASL6); K_2_CO_3_ (0.58 mmol) was added to the solution of *cis*-NO_2_-CA (0.29 mmol) in *N*-methyl-2-pyrrolidone (2.9 ml). 5-Bromo-3-methyl-2(5H)-franone (0.57 mmol) diluted in *N*-methyl-2-pyrrolidone (2.9 ml) was added to the mixture, and the mixture was stirred for 12 h at room temperature. The reaction was quenched by adding 1 N HCl, and the solution was diluted to 50 ml with water, extracted with EtOAc (3 × 50 ml). The organic layer was washed with distilled water (3 × 150 ml) and dried over Na_2_SO_4_ and concentrated *in vacuo*. The crude sample was purified by silica gel column chromatography (*n*-hexane/EtOAc:6/4) to afford *cis*-NO_2_-CASL (20.7 mg, 0.07 mol, 25%). ^1^H-NMR (300 MHz, CDCl_3_) 𝛿 8.23 (d, *J* = 8.7 Hz, 2 H), 7.71 (d, *J* = 8.7 Hz, 2 H), 7.18 (d, *J* = 12 Hz, 1 H), 6.89–6.88 (m, 2 H), 6.13 (d, *J* = 12 Hz, 1 H), 1.98 (s, 3 H); ^13^C-NMR (75 MHz, CDCl_3_), 𝛿 10.66, 92.42, 120.84, 123.29, 130.45, 134.70, 140.52, 141.63, 144.39, 147.93, 163.09, 170.89; HRMS [ESI+ (*m*/*z*)] calculated for (C_14_H_11_NO_6_ + H)^+^ 290.0659, found 290.0671.

*trans*-F-CASL (*t*-CASL7); K_2_CO_3_ (1.1 mmol) was added to the solution of *trans*-F-CA (0.57 mmol) in *N*-methyl-2-pyrrolidone (5.7 ml). 5-Bromo-3-methyl-2(5H)-franone (1.12 mmol) diluted in *N*-methyl-2-pyrrolidone (5.7 ml) was added to the mixture, and the mixture was stirred for 12 h at room temperature. The reaction was quenched by adding 1 N HCl, and the solution was diluted to 50 ml with water, extracted with EtOAc (3 × 50 ml). The organic layer was washed with distilled water (3 × 150 ml) and dried over Na_2_SO_4_ and concentrated *in vacuo*. The crude sample was purified by silica gel column chromatography (*n*-hexane/EtOAc:7/3) to afford *trans*-F-CASL (39.0 mg, 0.15 mol, 26%). ^1^H-NMR (300 MHz, CDCl_3_) 𝛿 7.75 (d, *J* = 16 Hz, 1 H), 7.56–7.51 (m, 2 H), 7.13–7.07 (m, 2 H), 7.02–6.98 (m, 2 H), 6.35 (d, *J* = 16 Hz, 1 H), 2.02 (t, *J* = 1.5 Hz, 3 H); ^13^C-NMR (75 MHz, CDCl_3_), 𝛿 10.59, 92.58, 115.65 (d, *J*_C-__*F*_ = 2.5 Hz), 116.15 (d, *J*_C-__*F*_ = 22 Hz), 129.99, 130.29 (d, *J*_C-__*F*_ = 8.6 Hz), 134.39, 142.12, 146.11, 164.23 (d, *J*_C-__*F*_ = 251 Hz), 164.69, 171.09; HRMS [ESI+ (*m*/*z*)] calculated for (C_14_H_11_FO_4_ + H)^+^ 263.0714, found 263.0744.

*cis*-F-CASL (*c*-CASL7); K_2_CO_3_ (1.26 mmol) was added to the solution of *cis*-F-CA (0.63 mmol) in *N*-methyl-2-pyrrolidone (6.3 ml). 5-Bromo-3-methyl-2(5H)-franone (1.23 mmol) diluted in *N*-methyl-2-pyrrolidone (6.3 ml) was added to the mixture, and the mixture was stirred for 12 h at room temperature. The reaction was quenched by adding 1 N HCl, and the solution was diluted to 50 ml with water, extracted with EtOAc (3 × 50 ml). The organic layer was washed with distilled water (3 × 150 ml) and dried over Na_2_SO_4_ and concentrated *in vacuo*. The crude sample was purified by silica gel column chromatography (*n*-hexane/EtOAc:7/3) to afford *cis*-F-CASL (94.2 mg, 0.36 mmol, 57%). ^1^H-NMR (300 MHz, CDCl_3_) 𝛿 7.72–7.67 (m, 2 H), 7.10–7.03 (m, 3 H), 6.92 (t, *J* = 1.5 Hz, 1 H), 6.89 (t, *J* = 1.5 Hz, 1H), 5.91 (d, *J* = 13 Hz, 1 H), 1.98 (t, *J* = 1.8 Hz, 3 H); ^13^C-NMR (75 MHz, CDCl_3_), 𝛿 10.56, 92.27, 115.14 (d, *J*_C-__*F*_ = 22 Hz), 116.73 (d, *J*_C-__*F*_ = 2.2 Hz), 130.11, 132.45 (d, *J*_C-__*F*_ = 8.7 Hz), 134.34, 141.97, 146.07, 163.34 (d, *J*_C-__*F*_ = 249 Hz), 163.65, 171.06; HRMS [ESI+ (*m*/*z*)] calculated for (C_14_H_11_FO_4_ + H)^+^ 263.0714, found 263.0734.

*trans*-Cl-CASL (*t*-CASL8); K_2_CO_3_ (1.0 mmol) was added to the solution of *trans*-Cl-CA (0.5 mmol) in *N*-methyl-2-pyrrolidone (5 ml). 5-Bromo-3-methyl-2(5H)-franone (0.98 mmol) diluted in *N*-methyl-2-pyrrolidone (5 ml) was added to the mixture, and the mixture was stirred for 12 h at room temperature. The reaction was quenched by adding 1 N HCl, and the solution was diluted to 50 ml with water, extracted with EtOAc (3 × 50 ml). The organic layer was washed with distilled water (3 × 150 ml) and dried over Na_2_SO_4_ and concentrated *in vacuo*. The crude sample was purified by silica gel column chromatography (*n*-hexane/EtOAc:7/3) to afford *trans*-Cl-CASL (128 mg, 0.46 mmol, 92%). ^1^H-NMR (300 MHz, CDCl_3_) 𝛿 7.73 (d, *J* = 16 Hz, 1 H), 7.48–7.27 (m, 4 H), 7.02 (t, *J* = 1.5 Hz, 1 H), 6.99 (t, *J* = 1.5 Hz, 1 H), 6.40 (d, *J* = 16 Hz, 1H), 2.02 (t, *J* = 1.5 Hz, 3 H); ^13^C-NMR (75 MHz, CDCl_3_), 𝛿 10.55, 92.55, 116.21, 129.42, 132.15, 134.34, 136.85, 142.08, 145.89, 164.53, 171.06; HRMS [ESI+ (*m*/*z*)] calculated for (C_14_H_11_ClO_4_ + H)^+^ 279.0419, found 279.0433.

*cis*-Cl-CASL (*c*-CASL8); K_2_CO_3_ (1.0 mmol) was added to the solution of *trans*-Cl-CA (0.52 mmol) in *N*-methyl-2-pyrrolidone (5 ml). 5-Bromo-3-methyl-2(5H)-franone (0.98 mmol) diluted in *N*-methyl-2-pyrrolidone (5 ml) was added to the mixture, and the mixture was stirred for 12 h at room temperature. The reaction was quenched by adding 1 N HCl, and the solution was diluted to 50 ml with water, extracted with EtOAc (3 × 50 ml). The organic layer was washed with distilled water (3 × 150 ml) and dried over Na_2_SO_4_ and concentrated *in vacuo*. The crude sample was purified by silica gel column chromatography (*n*-hexane/EtOAc:7/3) to afford *cis*-Cl-CASL (80.6 mg, 0.29 mmol, 56%). ^1^H-NMR (300 MHz, CDCl_3_) 𝛿 7.60 (d, *J* = 8.4 Hz, 2 H), 7.37–7.33 (m, 2 H), 7.05 (d, *J* = 13 Hz, 1 H), 6.92–6.88 (m, 2 H), 5.95 (d, *J* = 13 Hz, 1 H), 1.98 (t, *J* = 1.5 Hz, 3 H); ^13^C-NMR (75 MHz, CDCl_3_), 𝛿 10.55, 92.27, 117.64, 128.27, 131.45, 132.42, 134.34, 135.66, 141.91, 145.82, 163.52, 171.04; HRMS [ESI+ (*m*/*z*)] calculated for (C_14_H_11_ClO_4_ + H)^+^ 279.0419, found 279.0440.

*trans*-Br-CASL (*t*-CASL9); K_2_CO_3_ (1.0 mmol) was added to the solution of *trans*-Br-CA (0.5 mmol) in *N*-methyl-2-pyrrolidone (5 ml). 5-Bromo-3-methyl-2(5H)-franone (0.98 mmol) diluted in *N*-methyl-2-pyrrolidone (5 ml) was added to the mixture, and the mixture was stirred for 12 h at room temperature. The reaction was quenched by adding 1 N HCl, and the solution was diluted to 50 ml with water, extracted with EtOAc (3 × 50 ml). The organic layer was washed with distilled water (3 × 150 ml) and dried over Na_2_SO_4_ and concentrated *in vacuo*. The crude sample was purified by silica gel column chromatography (*n*-hexane/EtOAc:7/3) to afford *trans*-Br-CASL (157 mg, 0.49 mmol, 98%). ^1^H-NMR (300 MHz, CDCl_3_) 𝛿 7.71 (d, *J* = 16 Hz, 1 H), 7.54 (d, *J* = 8.4 Hz, 2 H), 7.39 (d, *J* = 8.4 Hz, 2 H), 7.01 (t, *J* = 1.5 Hz, 1 H), 6.98 (t, *J* = 1.5 Hz, 1H), 6.41 (d, *J* = 16 Hz, 1 H), 2.02 (t, *J* = 1.5 Hz, 3 H); ^13^C-NMR (75 MHz, CDCl_3_), 𝛿 10.59, 92.60, 116.59, 125.32, 129.63, 132.22, 132.61, 134.41, 142.05, 146.00, 164.55, 171.04; HRMS [ESI+ (*m*/*z*)] calculated for (C_14_H_11_BrO_4_ + H)^+^ 322.9913, found 322.9930.

*cis*-Br-CASL (*c*-CASL9); K_2_CO_3_ (0.56 mmol) was added to the solution of *cis*-Br-CA (0.28 mmol) in *N*-methyl-2-pyrrolidone (2.8 ml). 5-Bromo-3-methyl-2(5H)-franone (0.27 mmol) diluted in *N*-methyl-2-pyrrolidone (2.8 ml) was added to the mixture, and the mixture was stirred for 12 h at room temperature. The reaction was quenched by adding 1 N HCl, and the solution was diluted to 50 ml with water, extracted with EtOAc (3 × 50 ml). The organic layer was washed with distilled water (3 × 150 ml) and dried over Na_2_SO_4_ and concentrated *in vacuo*. The crude sample was purified by silica gel column chromatography (*n*-hexane/EtOAc:7/3) to afford *cis*-Br-CASL (34.8 mg, 0.11 mmol, 39%). ^1^H-NMR (300 MHz, CDCl_3_) 𝛿 7.56–7.48 (m, 4 H), 7.03 (d, *J* = 13 Hz, 1 H), 6.91 (t, *J* = 1.5 Hz, 1H), 6.88 (t, *J* = 1.5 Hz, 1 H), 5.96 (d, *J* = 13 Hz, 1 H), 1.98 (m, 3 H); ^13^C-NMR (75 MHz, CDCl_3_), 𝛿 10.60, 92.31, 117.86, 124.10, 131.30, 131.61, 132.93, 134.22, 141.87, 145.87, 163.53, 171.01; HRMS [ESI+ (*m*/*z*)] calculated for (C_14_H_11_BrO_4_ + H)^+^ 322.9913, found 322.9935.

*trans*-CF_3_-CASL (*t*-CASL10); K_2_CO_3_ (1.1 mmol) was added to the solution of *trans*-CF_3_-CA (0.56 mmol) in *N*-methyl-2-pyrrolidone (5.6 ml). 5-Bromo-3-methyl-2(5H)-franone (1.10 mmol) diluted in *N*-methyl-2-pyrrolidone (5.6 ml) was added to the mixture, and the mixture was stirred for 12 h at room temperature. The reaction was quenched by adding 1 N HCl, and the solution was diluted to 50 ml with water, extracted with EtOAc (3 × 50 ml). The organic layer was washed with distilled water (3 × 150 ml) and dried over Na_2_SO_4_ and concentrated *in vacuo*. The crude sample was purified by silica gel column chromatography (*n*-hexane/EtOAc:7/3) to afford *trans*-CF_3_-CASL (164 mg, 0.53 mmol, 94%). ^1^H-NMR (300 MHz, CDCl_3_) 𝛿 7.80 (d, *J* = 16 Hz, 1 H), 7.69–7.62 (m, 4 H), 7.03 (t, *J* = 1.5 Hz, 1 H), 6.99 (t, *J* = 1.5 Hz, 1 H), 6.50 (d, *J* = 16 Hz, 1 H), 2.03 (t, *J* = 1.5 Hz, 3 H); ^13^C-NMR (75 MHz, CDCl_3_), 𝛿 10.57, 92.65, 118.56, 123.63 (q, *J*_C-__*F*_ = 271 Hz), 125.90 (q, *J*_C-__*F*_ = 3.8 Hz), 128.41, 132.26 (q, *J*_C-__*F*_ = 33 Hz), 134.50, 137.05 (d, *J*_C-__*F*_ = 1.2 Hz), 141.96, 145.42, 164.28, 170.10; HRMS [ESI+ (*m*/*z*)] calculated for (C_15_H_12_F_3_O_4_ + H)^+^ 313.0682, found 313.0698.

*cis*-CF_3_-CASL (*c*-CASL10); K_2_CO_3_ (1.1 mmol) was added to the solution of *cis*-CF_3_-CA (0.54 mmol) in *N*-methyl-2-pyrrolidone (5.4 ml). 5-Bromo-3-methyl-2(5H)-franone (1.06 mmol) diluted in *N*-methyl-2-pyrrolidone 5.4 ml) was added to the mixture, and the mixture was stirred for 12 h at room temperature. The reaction was quenched by adding 1 N HCl, and the solution was diluted to 50 ml with water, extracted with EtOAc (3 × 50 ml). The organic layer was washed with distilled water (3 × 150 ml) and dried over Na_2_SO_4_ and concentrated *in vacuo*. The crude sample was purified by silica gel column chromatography (*n*-hexane/EtOAc:7/3) to afford *cis*-CF_3_-CASL (45.7 mg, 0.15 mmol, 27%). ^1^H-NMR (300 MHz, CDCl_3_) 𝛿 7.68–7.26 (m, 4 H), 8.16 (d, *J* = 12 Hz, 1 H), 6.89 (t, *J* = 1.4 Hz, 1 H), 6.85 (t, *J* = 1.4 Hz, 1 H), 6.06 (d, *J* = 13 Hz, 1 H), 1.97 (t, *J* = 1.4 Hz, 3 H); ^13^C-NMR (75 MHz, CDCl_3_), 𝛿 10.51, 92.28, 119.62, 123.83 (q, *J*_C-__*F*_ = 271 Hz) 124.95 (q, *J*_C-__*F*_ = 3.7 Hz), 129.84, 130.99 (q, *J*_C-__*F*_ = 32 Hz), 134.45, 137.69 (d, *J*_C-__*F*_ = 1.2 Hz), 141.73, 145.27, 163.29, 170.94; HRMS [ESI+ (*m*/*z*)] calculated for (C_15_H_12_F_3_O_4_ + H)^+^ 313.0682, found 313.0697.

*cis* and *trans* isomer mixture of the ethyl ester of indanone derived-CA analogs; Ethyl 2-(diethoxyphosphoryl) acetate (12.1 mmol) was added to a solution of NaH (60% oil, 15.2 mmol) in dry THF (12 ml) at −78°C under Ar. The mixture was stirred for 15 min, and then 1-indanone (10.1 mmol) in THF (6 ml) was added. After the stirring for 30 min, the mixture was placed on ice for 2 h and then stirred for 20 h at room temperature. The reaction was quenched with saturated aqueous NH_4_Cl and diluted with distilled water up to 100 ml. The mixture was extracted with ethyl acetate (3 × 100 ml) and the organic layer was washed with distilled water (2 × 300 ml). The organic layer was dried with Na_2_SO_4_, filtered, and concentrated *in vacuo*. The crude product was purified by silica gel column chromatography (*n*-hexane/EtOAc:9.5/0.5) to afford the ethyl ester of *trans*-indanone-CA (154.8 mg, 0.78 mmol, 8%) and *cis*-indanone-CA (58.4 mg, 0.29 mmol, 3%). ethyl ester of *trans*-indanone-CA: ^1^H-NMR (300 MHz, CDCl_3_) 𝛿 7.60 (d, *J* = 7.5 Hz, 1 H), 7.35 (d, *J* = 3.9 Hz, 2 H), (d, *J* = 7.8, 1 H), 7.23–7.28 (m, 1 H), 6.31 (t, *J* = 2.6 Hz, 1H), 4.23 (q, *J* = 7.2, 2 H), 3.28–3.33 (m, 2 H), 3.08 (t, *J* = 7.7 Hz, 1 H), 1.33 (t, *J* = 7.1 Hz, 3 H); ethyl ester of *cis*-indanone-CA: ^1^H-NMR (300 MHz, CDCl_3_) 𝛿 8.82 (d, *J* = 7.8 Hz, 1 H), 7.25–7.37 (m, 3 H), 5.97 (d, *J* = 2.0 Hz, 1 H), 4.22 (q, *J* = 7.2 Hz, 2 H), 2.97–3.01 (m, 2H), 2.89–2.94 (m, 2 H), 1.32 (t, *J* = 7.1 Hz, 2 H).

*trans*-indanone-CA; Ethanol (1.91 ml) and 5 M NaOHaq (1.93 ml) was added to the round flask containing *trans*-indanone-CA ethyl ester (0.77 mmol). The mixture was stirred for 15 h at room temperature. Distilled water was added to the solution up to 30 ml and extracted with ethyl acetate (3 × 30 ml). The pH of the water layer was adjusted to 1 using 6 N-HCl and, then extracted with ethyl acetate (3 × 30 ml). The organic layer was dried with Na_2_SO_4_, filtered, and concentrated *in vacuo*. The hydrolysis product was checked by TLC and used for the next coupling reaction without purification.

*trans*-indanone-CASL (*t*-indCASL); K_2_CO_3_ (0.91 mmol) was added to the crude *trans*-indanone-CA (0.45 mmol) in *N*-methyl-2-pyrrolidone (4.6 ml). 5-Bromo-3-methyl-2(5H)-franone (0.89 mmol) diluted in *N*-methyl-2-pyrrolidone (4.6 ml) was added to the mixture, and the mixture was stirred for 12 h at room temperature. The reaction was quenched by adding 1 N-HCl, and the solution was diluted to 50 ml with water, extracted with EtOAc (3 × 50 ml). The organic layer was washed with distilled water (3 × 150 ml) and dried over Na_2_SO_4_ and concentrated *in vacuo*. The crude sample was purified by a silica gel column chromatography (*n*-hexane/EtOAc:8/2) and PTLC (*n*-hexane/EtOAc:7/3) to afford *trans*-indanone-CASL (21.4 mg, 0.08 mmol, 18%). ^1^H-NMR (300 MHz, CDCl_3_) 𝛿 7.60 (d, *J* = 7.8 Hz, 1 H), 7.38–7.43 (m, 2 H), 7.25–7.30 (m, 1 H), 7.01 (quin, *J* = 1.5 Hz, 1 H), 6.97 (quin, *J* = 1.5 Hz, 2 H), 6.29 (t, *J* = 2.4 Hz, 1 H), 3.30–3.35 (m, 2 H), 3.11 (t, *J* = 5.9 Hz, 2 H), 2.01 (t, *J* = 1.5 Hz, 3 H); ^13^C-NMR (75 MHz, CDCl_3_), 𝛿 10.65, 30.57, 31.65, 92.24, 105.16, 121.90, 125.77, 126.93, 131.68, 134.18, 139.39, 142.49, 150.28, 165.12, 167.34, 171.36; HRMS [ESI+ (*m*/*z*)] calculated for (C_16_H_14_O_4_ + H)^+^ 271.0965, found 271.0978.

*cis*-indanone-CA; Ethanol (0.73 ml) and 5 M NaOHaq (0.73 ml) was added to the round flask containing *cis*-indanone-CA-Et (0.29 mmol). The mixture was stirred for 15 h at room temperature. Distilled water was added to the solution up to 30 ml and extracted with ethyl acetate (3 × 30 ml). The pH of the water layer was adjusted to 1 using 6 N-HCl and, then extracted with ethyl acetate (3 × 30 ml). The organic layer was dried with Na_2_SO_4_, filtered, and concentrated *in vacuo*. The hydrolysis product was checked by TLC and used for the next coupling reaction without purification.

*cis*-indanone-CASL (*c*-indCASL); K_2_CO_3_ (0.22 mmol) was added to the crude *cis*-indanone-CA (0.11 mmol) in *N*-methyl-2-pyrrolidone (1.1 ml). 5-Bromo-3-methyl-2(5H)-franone (0.22 mmol) diluted in *N*-methyl-2-pyrrolidone (1.1 ml) was added to the mixture, and the mixture was stirred for 12 h at room temperature. The reaction was quenched by adding 1 N-HCl, and the solution was diluted to 30 ml with water, extracted with EtOAc (3 × 30 ml). The organic layer was washed with distilled water (3 × 90 ml) and dried over Na_2_SO_4_ and concentrated *in vacuo*. The crude sample was purified by silica gel column chromatography (*n*-hexane/EtOAc:8/2) to afford *cis*-indanone-CASL (17.7 mg, 0.07 mmol, 60%). ^1^H-NMR (300 MHz, CDCl_3_) 𝛿 8.86 (d, *J* = 7.8 Hz, 1 H), 7.29–7.43 (m, 3 H), 6.98–6.99 (m, 1 H), 6.95 (quin, *J* = 1.7 Hz, 1 H), 5.94 (t, *J* = 1.8 Hz, 1 H), 6.29 (t, *J* = 2.4 Hz, 1 H), 2.93–3.0 (m, 4 H), 3.11 (t, *J* = 5.9 Hz, 2 H), 2.00 (t, *J* = 1.5 Hz, 3 H); ^13^C-NMR (75 MHz, CDCl_3_), 𝛿 10.68, 29.57, 36.03, 92.35, 108.14, 125.17, 126.79, 129.05, 131.65, 134.20, 137.15, 142.49, 151.78, 163.75, 165.45, 171.39; HRMS [ESI+ (*m*/*z*)] calculated for (C_16_H_14_O_4_ + H)^+^ 271.0965, found 271.097.

*cis* and *trans* isomer mixture of the ethyl ester of tetralone derived-CA analogs; Ethyl 2-(diethoxyphosphoryl) acetate (12.08 mmol) was added to a solution of NaH (60% oil, 15.15 mmol) in dry THF (12 ml) at −78°C under Ar. The mixture was stirred for 15 min, and then 1-tetralone (10 mmol) in THF (6 ml) was added. After the stirring for 30 min, the mixture was placed on ice for 16 h and then stirred for 2 h at room temperature. The reaction was quenched with saturated aqueous NH_4_Cl and diluted with distilled water up to 50 ml. The mixture was extracted with dichloromethane (3 × 50 ml). The Organic layer was dried with Na_2_SO_4_, filtered, and concentrated *in vacuo*. The crude product was purified by silica gel column chromatography (*n*-hexane/EtOAc:9/1) to afford the mixture of *trans*-tetralone-CA-Et and *cis*-tetralone-CA-Et. The mixture was used for the next hydrolysis reaction without purification.

*cis* and *trans* isomer mixture of tetralone derived-CA analogs; Ethanol (0.74 ml) and 5 M NaOHaq (0.75 ml) was added to the round flask containing the mixture of *trans*-tetralone-CA-Et and *cis*-tetralone-CA-Et (0.30 mmol). The mixture was stirred for 17 h at room temperature. Distilled water was added to the solution up to 30 ml and extracted with ethyl acetate (3 × 30 ml). Water phase pH was adjusted to 1 using 6 N-HCl and then extracted with ethyl acetate (3 × 30 ml). The organic layer was dried with Na_2_SO_4_, filtered, and concentrated *in vacuo*. These hydrolysis products were checked by TLC and used for the next coupling reaction without purification.

*trans*-tetralone-CASL (*t*-tetCASL) and *cis*-tetralone-CASL (*c*-tetCASL); K_2_CO_3_ (0.39 mmol) was added to the mixture of *trans*-tetralone-CA and *cis*-tetralone-CA (0.19 mmol) in *N*-methyl-2-pyrrolidone (1.9 ml). 5-Bromo-3-methyl-2(5H)-franone (0.38 mmol) diluted in *N*-methyl-2-pyrrolidone (1.9 ml) was added to the mixture, and the mixture was stirred for 12 h at room temperature. The reaction was quenched by adding 1 N-HCl, and the solution was diluted to 30 ml with water, extracted with EtOAc (3 × 30 ml). The organic layer was washed with distilled water (3 × 90 ml) and dried over Na_2_SO_4_ and concentrated *in vacuo*. The crude sample was purified by silica gel column chromatography (*n*-hexane/EtOAc:8/2) to afford *trans*-tetralone-CASL (21.4 mg, 0.08 mmol, 40%) and *cis*-tetralone-CASL. *cis*-tetralone-CASL was purified again by reverse phase HPLC (ODS-SP100, CH_3_CN/H_2_O:7/3; 8.6 mg, 0.03 mmol, 16%). *trans*-tetralone-CASL: ^1^H-NMR (300 MHz, CDCl_3_) 𝛿 7.64 (d, *J* = 7.8 Hz, 1 H), 7.16–7.34 (m, 3 H), 6.99 (s, 1 H), 6.96 (s, 1 H), 6.31 (s, 1 H), 3.20–3.22 (m, 2 H), 2.81 (t, *J* = 6.0 Hz, 2 H), 2.01 (t, *J* = 1.5 Hz, 3 H), 1.87 (t, *J* = 6.0 Hz, 3 H); ^13^C-NMR (75 MHz, CDCl_3_), 𝛿 10.69, 22.47, 28.57, 30.10, 92.18, 109.52, 124.93, 126.51, 129.29, 130.56, 133.59, 134.24, 140.85, 142.48, 159.19, 164.53, 171.37; HRMS [ESI+ (m/z)] calculated for (C_17_H_16_O_4_ + H)^+^ 285.1121, found 285.1134; *cis*-tetralone-CASL: ^1^H-NMR (300 MHz, CDCl_3_) 𝛿 7.65 (d, *J* = 7.8 Hz, 1 H), 7.27–7.32 (m, 1 H), 7.13–7.18 (m, 2 H), 6.93 (t, *J* = 1.5 Hz, 1 H), 6.89 (t, *J* = 1.5 Hz, 1 H), 5.78 (s, 1 H), 2.87 (t, *J* = 6.6 Hz, 2 H), 2.52–2.56 (m, 2 H), 1.96–2.02 (m, 5 H); ^13^C-NMR (75 MHz, CDCl_3_), 𝛿 10.66, 22.99, 29.09, 35.42, 92.29, 111.88, 124.85, 128.42, 129.75, 130.21, 132.61, 134.20, 139.42, 142.28, 158.45, 164.41, 171.34; HRMS [ESI+ (*m*/*z*)] calculated for (C_17_H_16_O_4_ + H)^+^ 285.1121, found 285.1132.

GR5; γ-butyrolactone (2.0 mmol) and methyl formate (10 mmol) was added to the solution of potassium *tert* -butoxide in dry tetrahydrofuran at 4°C under Ar. The mixture was transferred to the room temperature and stirred for 6 h. 5-Bromo-3-methyl-2(5H)-franone (2.0 mmol) was added and stirred for 15 h at room temperature. The reaction was quenched by adding 1 N HCl, and the solution was diluted to 100 ml with distilled water, extracted with EtOAc (3 × 100 ml). The organic layer was dried over Na_2_SO_4_ and concentrated *in vacuo*. The crude sample was purified by silica gel column chromatography (*n*-hexane/EtOAc:5/5). Fractions containing the GR5 were purified again by silica gel column chromatography (Toluene/EtOAc:6/4; 46.2 mg, 0.22 mmol, 11%). ^1^H-NMR (300 MHz, CDCl_3_) 𝛿 7.48 (t, *J* = 2.7 Hz, 1 H), 6.94 (quin, *J* = 1.5 Hz, 1 H), 6.17(t, *J* = 1.5 Hz, 1 H), 4.39 (t, *J* = 7.2 Hz, 2 H), 2.93–2.87 (m, 2 H), 2.03 (t, *J* = 1.5 Hz, 3 H).

### Germination Assay (*Orobanche minor* and *Striga hermonthica*)

*Orobanche minor* seeds were washed with 70% EtOH, and then sonicated for 4 min in 1% sodium hypochlorite solution containing 0.2% Tween-20. The seeds were then washed 10 times with sterile water and suspended in 0.1% agar solution. The seeds were loaded onto 5-mm glass fiber filter disks (20–70 seeds/disk) and were conditioned at 23°C for 15 days. Each disk was transferred into a 96-well plate. A 30 μl aliquot of test chemical solution was added to the well. For the germination assay, the chemical solutions were prepared by 1,000 times dilution from each acetone stock solution with water (final acetone concentration was 0.1%). GR24 solution (0.1% acetone) and sterile water (0.1% acetone) was used as positive and negative control, respectively. The 96 well plates were incubated for 5 days at 23°C and the number of total seeds and germinated seeds was counted.

*Striga hermonthica* seeds were washed with 70% EtOH, and then sonicated for 4 min in 1% sodium hypochlorite solution containing 0.2% Tween-20. The seeds were then washed 10 times with sterile water and suspended in 0.1% agar solution. The seeds were loaded onto 5-mm glass fiber filter disks (20–70 seeds/disk) and were conditioned at 30°C for 7 days. Each disk was transferred into a 96-well plate. A 30 μl aliquot of test chemical solution was added to the well. For the germination assay, the chemical solutions were prepared by 1,000 times dilution from each acetone stock solution with water (final acetone concentration was 0.1%). GR24 solution (0.1% acetone) and sterile water (0.1% acetone) was used as positive and negative control, respectively. The 96 well plates were incubated for 1 day at 30°C and total seeds and germinated seeds were counted.

### Protein Expression (ShHTL6, ShHTL7, and AtD14)

The ORF fragment of ShHTL6 and ShHTL7 was synthesized using IDT gBlock service. The PCR amplified ShHTL6 and ShHTL7 fragment was digested with Nco I and EcoR I, and the digested product was introduced into a modified pET28 vector with an N-terminal His8 tag. *Escherichia coli* BL21(DE3) was used for protein expression. The overnight culture (12 ml) was added to a fresh LB medium (1.2 L) containing kanamycin (50 mg/L) at 37°C. After OD_600_ reached 0.8, 0.1 mM IPTG was added and the cell was further incubated at 16°C for 21 h. The culture medium was centrifuged at 3700 *g* and the pellet was stored at −20°C until use. The pellet was resuspended and sonicated in a lysis buffer (50 mM Tris buffer (pH 8.0) containing 500 mM NaCl, 10 mM 2-mercaptoethanol, and 10% glycerol). The supernatant was purified by Ni Sepharose TM 6 Fast Flow (500 μl, Cytiva). After washing with the washing buffer (50 mM Tris buffer (pH 8.0) containing 500 mM NaCl and 20 mM imidazole), The bound protein was eluted with elution buffer (50 mM Tris buffer (pH 8.0) containing 500 mM NaCl and 200 mM imidazole). The eluate was concentrated using VIVASPIN Turbo 15 (Sartorius), and the concentration was adjusted to 5 mg/ml. The purified protein was aliquoted to the appropriate volume, immediately frozen in liquid nitrogen, and stored at −80°C until use.

The cDNA of AtD14 was introduced into pMALHis vector that has both MBP-tag and His6-tag. The vector was transformed in to *E. coli* BL21 (DE3) and the cell was precultured in LB medium containing 50 μg/ml ampicillin. Overnight cultures (10 ml) were added to fresh LB medium (1 L) containing 50 μg/ml ampicillin and it was cultured at 37°C. After OD_600_ reached to 0.8, the cultures were cooled at 16°C for 1 h, and then 0.1 mM IPTG was added. The culture was further incubated at 16°C for 20 h. The culture medium was centrifuged at 3,700 *g* and the pellet was stored at −20°C until use. The pellet was resuspended and sonicated in lysis buffer (50 mM Tris buffer (pH 8.0) containing 500 mM NaCl, 10 mM 2-mercaptoethanol, and 10% glycerol). The supernatant was purified by Ni Sepharose TM 6 Fast Flow (500 μl, Cytiva) After washing with the washing buffer (50 mM Tris buffer (pH 8.0) containing 500 mM NaCl and 20 mM imidazole), The bound protein was eluted with elution buffer (50 mM Tris buffer (pH 8.0) containing 500 mM NaCl and 200 mM imidazole). The eluate was concentrated using VIVASPIN Turbo 15 (Sartorius), and the concentration was adjusted to 5 mg/ml. The purified protein was aliquoted to the appropriate volume, immediately frozen in liquid nitrogen, and stored at −80°C until use.

### YLG Assay

*In vitro* YLG assays were conducted using 1 μg of recombinant ShHTL6 or ShHTL7 in 100 μl of reaction buffer (100 mM HEPES, 150 mM NaCl, pH 7.0) with 0.2% DMSO on a 96-well black plate (Greiner). After YLG was incubated with recombinant ShHTL6 or ShHTL7 for 1 h, the fluorescence was measured by spectraMax i5 (Molecular Devices) at excitation by 480 nm and detection by 520 nm. IC_50_ values were calculated using the online tool Quest GraphTM IC_50_ Calculator (AAT Bioquest, Inc., United States; https://www.aatbio.com/tools/ ic50-calculator).

### Branching Assay

Sterilized seeds of the Arabidopsis *max4-8* mutant were put on the rockwool which was soaked in Noren’s hydroponic solution (Noren et al., 2004) and cultured at 22°C for 14 days under LED light (105 μmol/m^2^/s) with a 16 h light/8 h dark photoperiod. Seedlings were transferred to a plastic pot containing 800 ml of hydroponic culture and grown under the same conditions for additional 15 or 16 days. Test compounds were dissolved in acetone and added to hydroponic culture. The final concentration of test compounds was 1 or 5 μM and acetone was adjusted to 0.01% (v/v). The hydroponic culture was renewed after 7 days. The number of rosette branches (>5 mm) was counted. In the experience at 5 μM concentration, fresh weight of shoot was also measured to evaluate the side effects of tested compounds.

### Differential Scanning Fluorometry Experiments

DSF was conducted using 10 μg of the recombinant AtD14 protein in 20 μl of PBS buffer containing the 0.02 μl of Sypro Orange (Ex/Em: 490/610 nm; Invitrogen) and each compound with 5% (v/v) acetone on 96-well plate. These mixtures were heated from 20°C to 95°C, and the fluorescence (Ex/Em; 483/568) was constantly scanned by using LightCycler480 (Roche). The denaturation curve was calculated by using the LightCycler480 Software.

### Hydrolysis Assay

Hydrolysis assays were carried out at 30°C for 60 min in 100 μl of a reaction buffer containing 0.13 pmol recombinant ShHTL7 or AtD14, 10 μM of substrate in 50 mM Phosphate-Na buffer (pH 7.0) containing 2% acetone. The enzyme reaction was stopped by addition of 100 μl of acetonitrile containing 1-naphtaleneacetic acid (NAA; 0.5 ng/μl) as an internal standard. After centrifuged at 13,000 rpm for 5 min, each sample was subjected to LC–MS/MS analysis equipped with reverse-phase column (CORTECS UPLC Phenyl 1.6 μm, ϕ2.1 × 75 mm; Waters). The concentration of remaining substrate and reaction product was calculated by using NAA as an internal standard. Detailed information about the analytical condition is described in Supplementary Table 1.

### Time Course Monitoring of CASLs Degradation in Hydroponic Culture

*t*-CASL1, *c*-CASL1, PPASL, and GR5 were separately added to 15 ml tubes containing hydroponic culture. The final concentration of the test compounds is 1 μM and acetone was adjusted to 0.01% (v/v). These solutions were incubated under the same conditions as branching assay. After incubation for 0, 5, 24 72, or 168 h, 495 μl of hydroponic culture containing test compounds was collected and 5 μl of NAA solution (10 μM) was added as an internal standard. The solution was extracted with ethyl acetate. The ethyl acetate phase was dried up under the nitrogen gas and dissolved in acetonitrile. These samples were subjected to LC–MS/MS analysis equipped with reverse-phase column (CORTECS UPLC Phenyl 1.6 μm, ϕ2.1 × 75 mm; Waters). Each peak area of test chemicals and internal standard was calculated, and the remaining percentage of teste chemicals was calculated. At the same time, the degradation products of test compounds (CA or PPA) were also analyzed. Detailed information about the analytical condition was described in Supplementary Table 1.

### Growth Promoting Assay and Root Phenotypic Analysis

Sterilized seeds of the *Arabidopsis* wild type Col-0 were put on the plate containing 1% (w/v) agar-solidified 0.5× Murashige and Skoog (MS) medium, 1% sucrose. Test compounds were dissolved in the medium at 1 μM by 10,000 times dilution from each acetone stock (final concentration of acetone was 0.01% (v/v)). To evaluate the growth promoting effect, these seedlings were cultured horizontally at 22°C for 20 days under LED light (105 μmol/m^2^/s) with a 16 h light/8 h dark photoperiod. After the cultivation, flesh weight of shoot was measured. For root phenotypic analysis, these seedlings were cultured verticaly at 22°C for 11 days under LED light (105 μmol/m^2^/s) with a 16 h light/8 h dark photoperiod. After the cultivation, root length was measured by using ImageJ software and number of lateral roots was counted by using a microscope.

## Results

### Synthesis of SL Analogs Derived From CAs

**Figure 2:**
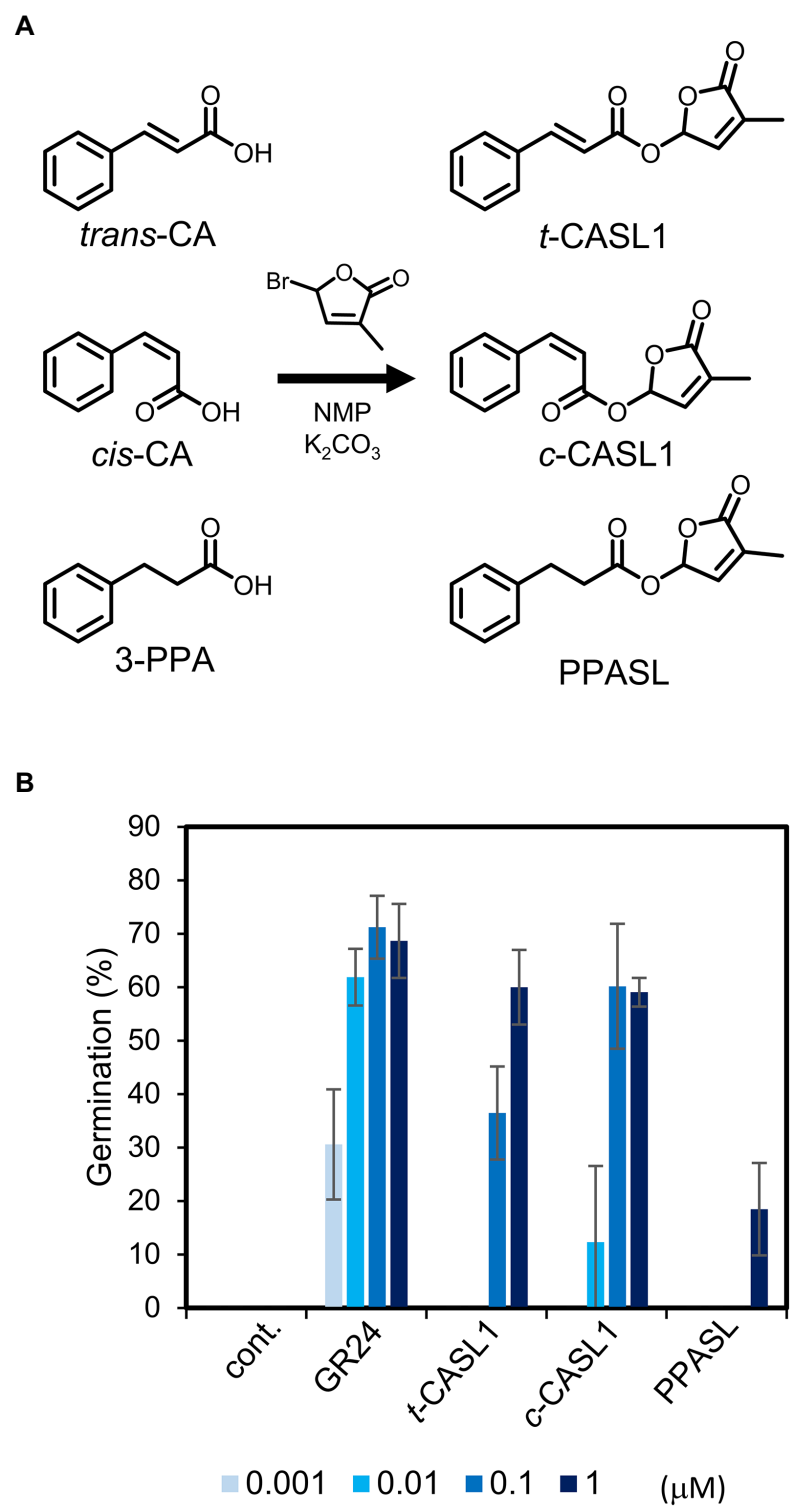
*Orobanche minor* germination inducing activity of cinnamic acid (CA) derived SL analogs. **(A)** Synthetic scheme for the CA derived SL analogs. **(B)** Germination inducing activity of CASLs toward *O. minor* seeds. Data are the means ± SD (*n* = 3). cont. means control with only acetone at 0.1%.

To develop new SL analogs derived from cinnamic acid, we simply coupled the methylbutenolide (D-ring) to the carboxylic acid part of CA. To obtain structurally diverse analogs, we used as the starting materials both *trans* and *cis* CA isomers, and also 3-phenylpropionic acid (3-PPA), in which the double bond in the side chain is reduced to a single bond (Figure 2). It was previously reported that the isomerization of *trans*-CA to *cis*-CA is promoted by UV irradiation (254 nm; Yang et al., 1999). Accordingly, we prepared *cis*-CA according to this reported method. UV irradiation of the commercial *trans*-CA in MeOH solution promoted isomerization to the *cis*-isomer. Only the *cis*-isomer could be dissolved with water. Therefore, we could easily collect the *cis*-isomer after the isomerization, and it was then subjected to the D-ring coupling reaction. Hereafter, these new SL analogs derived from the isomers pair are referred to as *c*-CASL for *cis* isomer and *t*-CASL for *trans* isomer, respectively. The 3-PPA-derived SL analog is referred to as PPASL (Figure 2). Using these three analogs (*c*-CASL1, *t*-CASL1, and PPASL), we tested their germination-inducing activity for seeds of a root parasitic plant, *Orobanche minor*. We found that *c*-CASL1 showed approximately 10 times stronger germination-inducing activity than *t*-CASL1. Moreover, PPASL showed much weaker activity compared with *c*-CASL1 or *t*-CASL1, suggesting that the presence of the double bond in the side-chain structure has an important role for the biological activity as germination inducer for *O. minor*.

### Synthesis of C-4 Substituted CASLs and Conformationally Fixed Analogs

**Figure 3:**
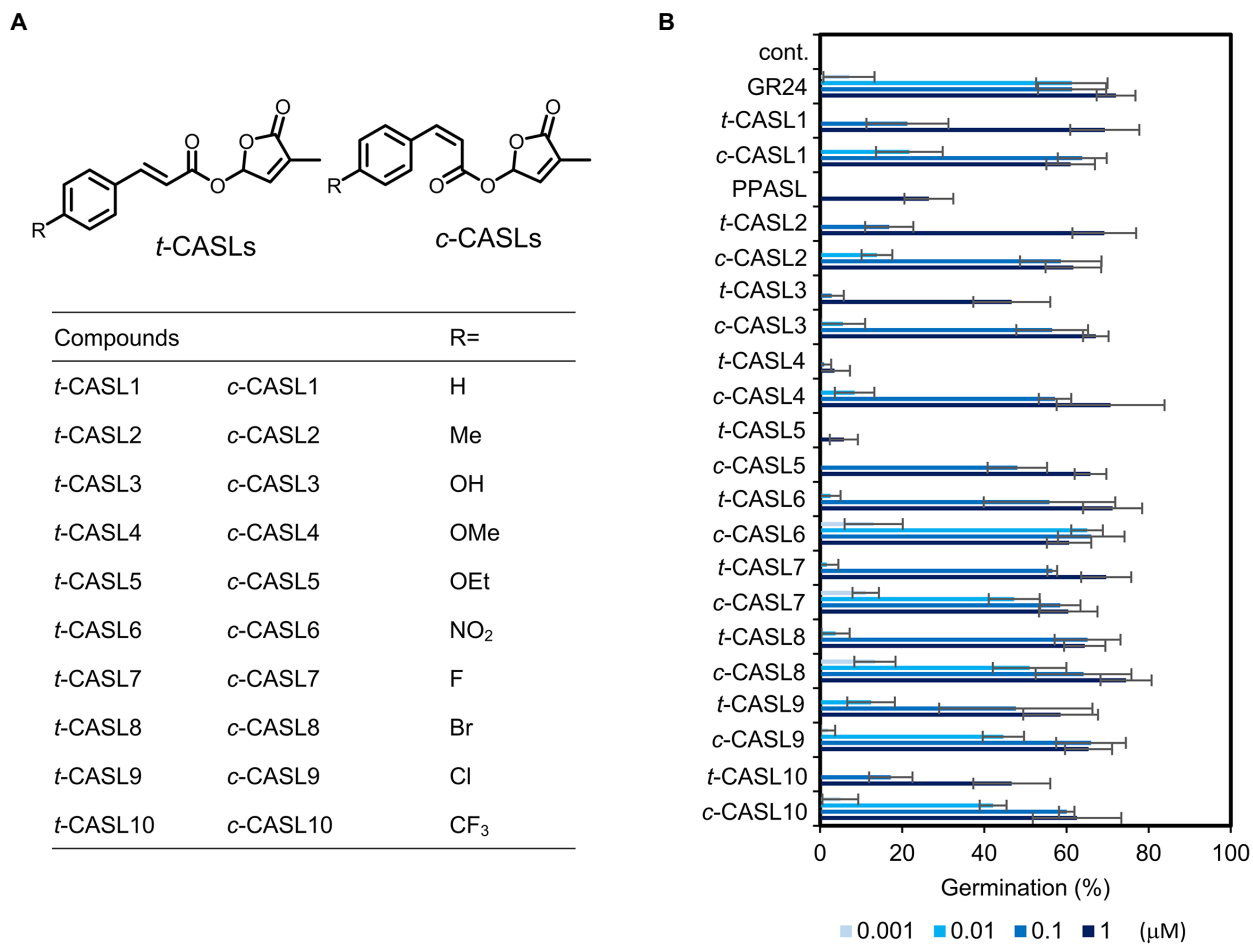
*Orobanche minor* germination inducing activity of C-4 substituted CA derived SL analogs. **(A)** Chemical structures of C-4 substituted type CASLs. **(B)** Germination inducing activity of tested compounds toward *O. minor* seeds. Data are the means ± SD (*n* = 3). cont. means control with only acetone at 0.1%.

To obtain more potent analogs derived from CA, we further synthesized structurally diverse CASLs using 4-substituted CA derivatives as the starting materials. Each *cis*-isomer of CA was obtained by the above-mentioned method, and in total we synthesized 18 analogs (*c*-CASL2 to *c*-CASL10, *t*-CASL2 to *t*-CASL10, Figure 3A). Among these SL analogs, interestingly, *c*-CASL type isomers showed stronger activity compared with the corresponding *trans* isomer, with the approximately 10 times activity difference. Moreover, we found that introduction of an electron withdrawing group, such as NO_2_, CF_3_, Br, Cl, increased the germination-inducing activity with almost 10 times strength compared with the non-substituted compounds. These compounds induced *O. minor* germination at 1 nM concentration, which was almost equal activity to the positive control, GR24 (Figure 3B).

**Figure 4:**
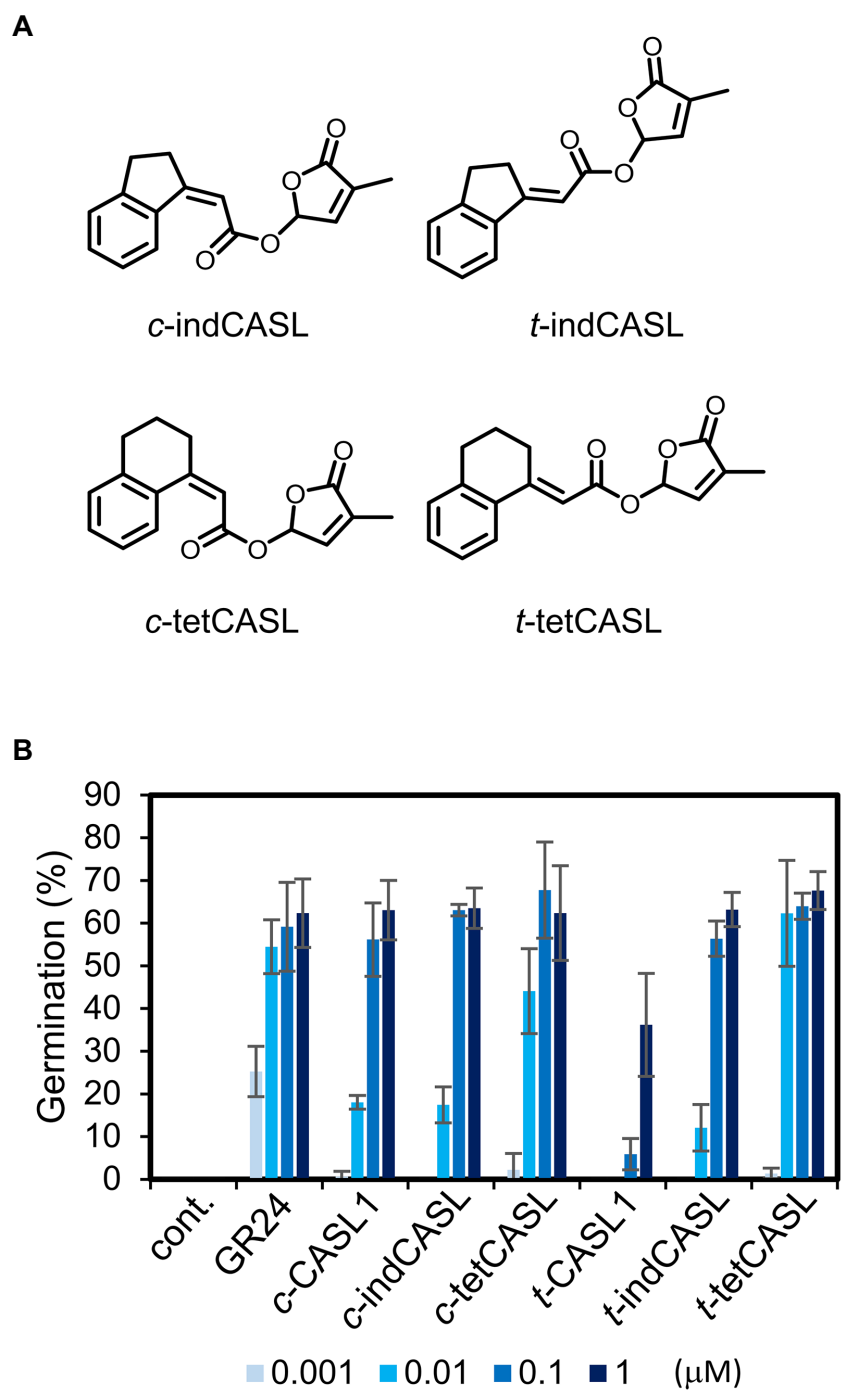
*Orobanche minor* germination inducing activity of conformationally fixed CA derived SL analogs. **(A)** Chemical structures of the conformationally fixed CASLs. **(B)** Germination inducing activity of tested compounds toward *O. minor* seeds. Data are means ± SD (*n* = 3). cont. means control with only acetone at 0.1%.

We found that *c*-CASLs showed stronger activity for inducing *O. minor* germination than *t*-CASLs. In these synthetic analogs, the side-chain structure has flexible conformation because of its free rotation. In a previous study, this rotation of the CA side-chain was fixed by introducing a ring structure to connect the benzene ring to the side-chain double bond. Such conformationally restricted *cis*-CA analogs were found to have stronger activity as allelochemicals than non-fixed analogs (Nishikawa et al., 2013). Thus, we also prepared conformationally-fixed CASL analogs. Starting from indanone or tetralone, we introduced an olefin part by the Horner–Wadsworth–Emmons reaction, which produced a mixture of both *cis* and *trans* isomers of conformationally-fixed CA analogs. After hydrolysis of the ethyl ester, they were further subjected to the D-ring coupling reaction, which yielded conformationally restricted *c*-CASL and *t*-CASL (Figure 4A; indCASL or tetCASL). We found that both *c*-indCASL and *c*-tetCASL showed slightly stronger activity for inducing *O. minor* germination, compared with the corresponding unfixed analog (Figure 4B). In contrast, both *t*-indCASL and *t*-tetCASL showed much stronger activity, compared with the non-fixed analog, *t*-CASL1 (Figure 4B). These *trans* analogs showed activity almost equal to the corresponding *cis* isomer, showing that the activity difference between *cis* and *trans* isomer decreased by fixing the side chain rotation.

### Interaction of CA-Derived SLs and the SL Receptor Protein

**Figure 5:**
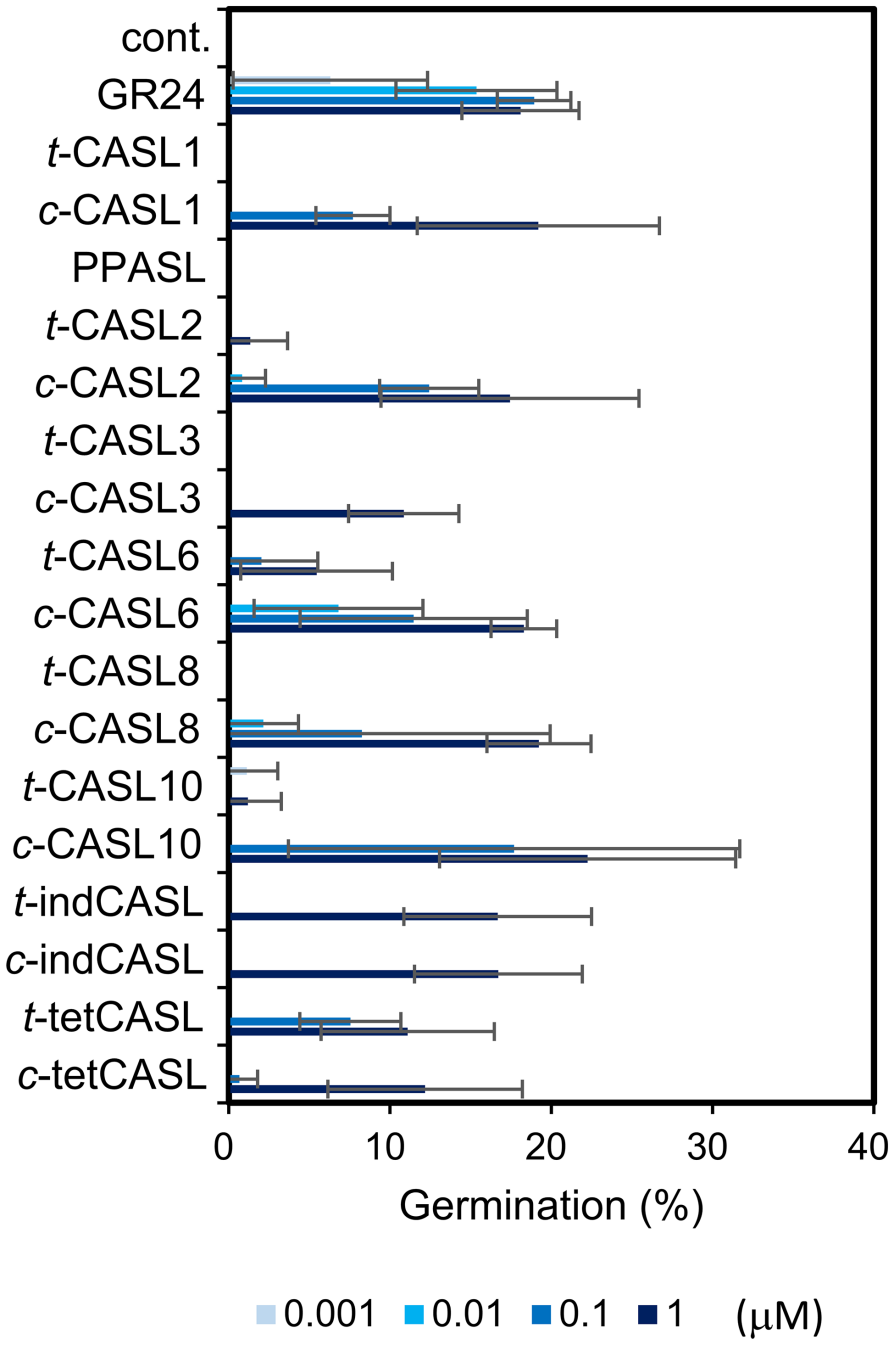
Germination inducing activity of CASLs toward *Striga hermonthica* seeds. Data are the means ± SD (*n* = 3). cont. means control with only acetone at 0.01%.

As mentioned above, we found that *cis*-CA-derived SL analogs showed stronger activity for inducing *O. minor* germination. To understand the molecular basis of the difference in activity between *cis* and *trans* isomers, we examined the interaction of each analog with the SL receptor protein. Because the SL receptors in *O. minor* have not yet been identified, we used the *S. hermonthica* sensitive receptor, HTL7 (Toh et al., 2015). We tested the interaction between HTL7 and each SL analog using yoshimulactone green (YLG), a pro-fluorescence-type SL analog. As mentioned earlier, HTL7 is a member of α/β-fold hydrolase family, and it was reported that YLG is hydrolysable by HTL7. YLG hydrolysis releases the fluorescein molecule, which can be easily monitored by fluorescence detector (Tsuchiya et al., 2018). As was reported previously, the SL analog, GR24, effectively inhibited the fluorescence emission with IC_50_ value 0.17 μM (Table 1; Supplementary Figure 1). Similarly, we found that the CASLs also inhibited YLG hydrolysis by HTL7 with moderately low IC_50_ values. Interestingly, *c*-CASLs showed lower IC_50_ values, compared with the corresponding *t*-CASLs (Table 1; Supplementary Figure 1). We also tested the interaction of these analogs with another SL receptor, HTL6, which is close to HTL7 but is not so sensitive compared with HTL7. Even in the case of HTL6, *c*-CASL showed lower IC_50_ value compared with *t*-CASL or PPASL (Supplementary Figure 2). These results strongly suggested that the activity difference between the *cis* and *trans* isomers of CASLs was because of the affinity difference for the receptor protein. As mentioned above, PPASL showed weaker activity for inducing *O. minor* germination, but this analog also inhibited YLG hydrolysis by HTL7 or HTL6, with a moderate IC_50_ value. Because we were able to detect the direct interaction of CASLs with the *Striga* SL receptor, we tested the germination-inducing activity of these compounds toward *S. hermonthica* seeds. We found that CASLs induced the germination of *S. hermonthica* seeds at moderately low concentration, and the activity of *c*-CASLs was stronger than that of the corresponding *t*-CASL, as was the case with *O. minor* (Figure 5).

**Table 1:** Evaluation of me direct interaction between ShHTL7 and CASLs.

Compounds	IC_50_ (μM)	Compounds	IC_50_ (μM)
GR24	0.17	PPASL	1.46
*t*-CASL1	2.05	*c*-CASL1	0.59
*t*-CASL2	1.41	*c*-CASL2	0.68
*t*-CASL3	>10	*c*-CASL3	3.43
*t*-CASL4	2.21	*c*-CASL4	0.66
*t*-CASLS	3.97	*c*-CASLS	0.31
*t*-CASL6	0.71	*c*-CASL6	0.25
*t*-CASL7	1.55	*c*-CASL7	1.14
*t*-CASL8	0.70	*c*-CASL8	0.36
*t*-CASL9	0.83	*c*-CASL9	0.34
*t*-CASL10	0.34	*c*-CASL10	0.22
*t*-indCASL	3.07	*c*-indCASL	2.01
*t*-tetCASL	3.85	*c*-tetCASL	4.61

IC_50_ values for the tested compounds in YLG assay were calculated using the online tool Quest GraphTM IC_50_ Calculator (AAT Bioquest, Inc., United States).

### Shoot-Branching Inhibition Activity by CASLs

**Figure 6:**
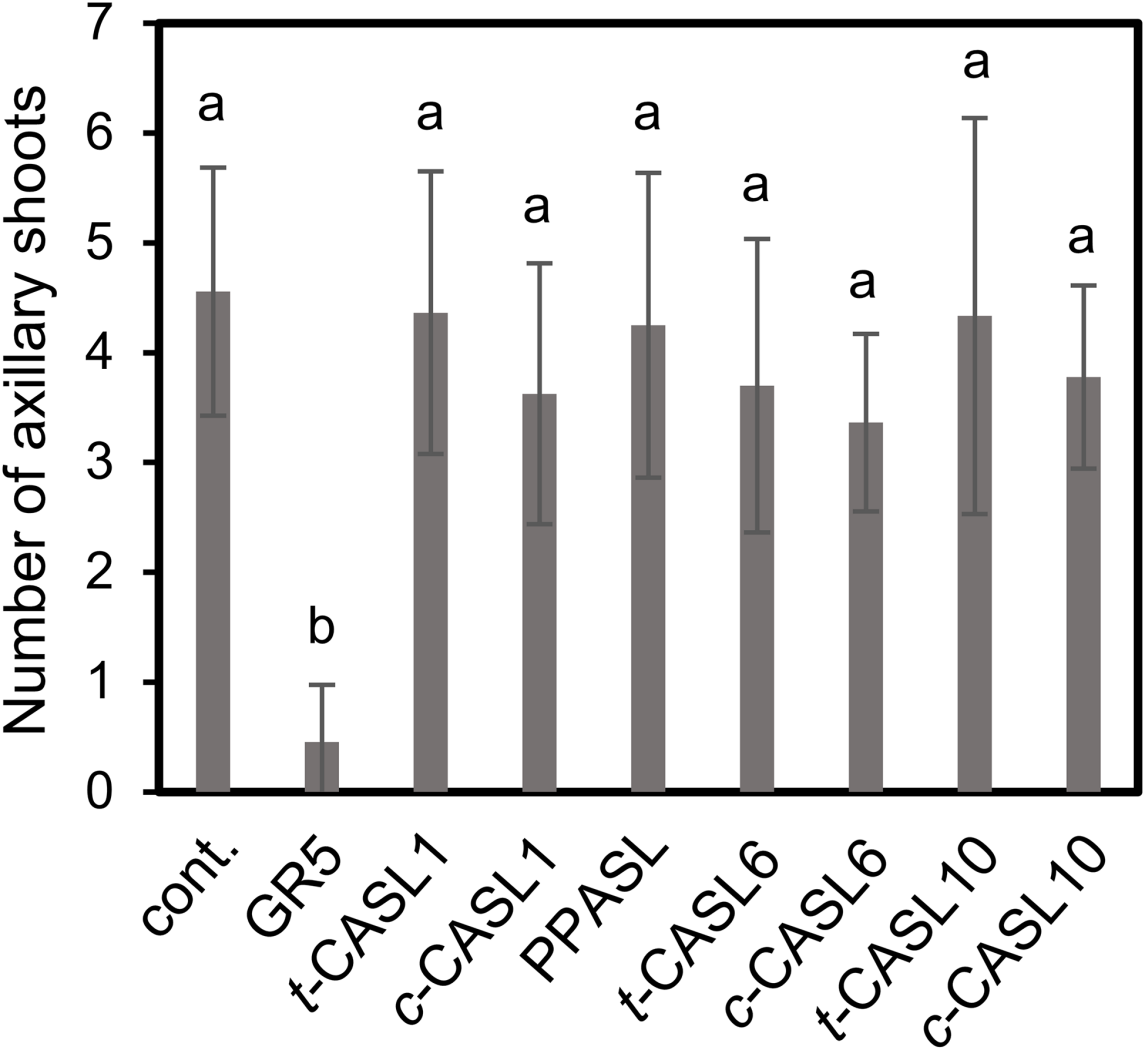
Shoot branching inhibiting activity of CASLs using the Arabidosis *max4* mutant. Number of axillary shoots (Over 5 mm) of *max4* which were grown in hydroponic culture containing test compounds at 1 μM concentration was counted. Data are means ± SD (*n* = 8–11). Different letters indicate significant differences at *p* < 0.05 with Tukey multiple comparison test. cont. means control with only acetone at 0.1%.

We next evaluated the activity of CASLs as plant hormones using a shoot-branching inhibition assay. The *Arabidopsis* SL biosynthetic mutant, *max4*, was hydroponically grown in the presence of each synthetic CASL analog. As was reported previously, 1 μM GR5, a simplified SL analog, clearly inhibited shoot branching of the *max4* mutant (Umehara et al., 2015), but we did not see shoot-branching inhibition by any of these CASLs at the same concentration (Figure 6). At a higher concentration, 5 μM, we found slight decrease of the branching number by treatment with some of synthetic analogs. However, we also found that such chemicals also showed growth inhibiting activity with significant decrease of shoot weight, which might be the reason for the branching reduction (Supplementary Figure 3). Next, we examined the direct interaction of the *Arabidopsis* SL receptor, AtD14, with CASLs, using differential scanning fluorimetry (DSF), which is widely used for SL receptor biochemical analyses (Hamiaux et al., 2012; Seto et al., 2019). This method can evaluate the receptor–ligand interaction by measuring the ligand-inducible melting temperature shift of the receptor protein. Using this method, we found that both *cis-* and *trans*-CASLs induced a melting temperature shift of AtD14, suggesting that these CASLs were able to interact directly with the *Arabidopsis* SL receptor protein, at least *in vitro* (Supplementary Figure 4). To further address the weak activity of CASLs in the shoot branching inhibition assay, we examined the chemical stability of CASLs in the hydroponic culture conditions. We speculated that these analogs might be unstable because the D-ring is connected to the CA part by an ester bond. We simply incubated *c*-CASL1, *t*-CASL1, or PPASL in the hydroponic culture medium, which was used for the shoot-branching inhibition assay, and the non-enzymatic degradation of each chemical was monitored by LC–MS/MS analysis. As we expected, both *c*-CASL1 and *t*-CASL1 degraded more rapidly than did GR5, with almost 40% loss within 1 week (Supplementary Figure 5A). In addition, we detected the release of the CA part, which gradually increased over time (Supplementary Figure 5B). Thus, instability of these analogs could be one reason for the inactivity of CASLs in the shoot-branching inhibition assay. We also found that SL receptors, AtD14 and HTL7, can hydrolyze *c*-CASL, *t*-CASL, or PPASL (Supplementary Figure 6). Thus, receptor-dependent degradation of these compounds might be also the reason for weak activity in shoot branching inhibition. However, we cannot rule out some other possibilities, for instance that the uptake of these analogs by *Arabidopsis* might be quite poor for some reason, or that CASLs might be metabolized rapidly *in planta*. Moreover, as mentioned above, CASLs exhibited growth inhibiting activity toward Arabidopsis at a high concentration. Thus, this would be another possible reason for apparent weak activity of CASLs as plant hormones.

### Plant Growth Promoting Activity of CASLs

**Figure 7:**
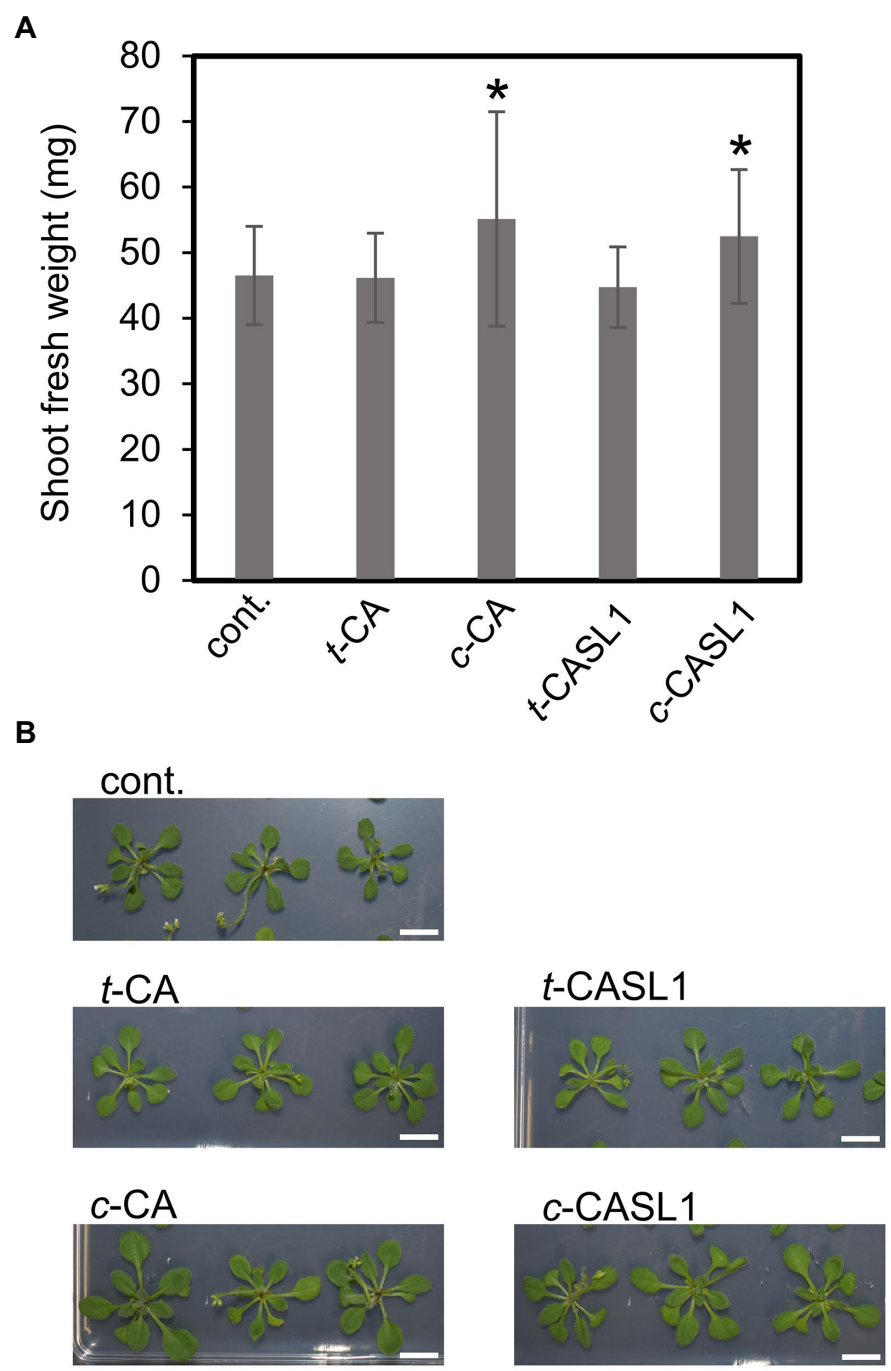
Effects of the CAs and CASLs on the *Arabidosis* seedling growth. **(A)** Fresh weight of the shoot parts after growing for 20 days on agar plate containing 1 μM test compounds. Data are the means ± SD (*n* = 13–25). Asterisk indicate the significant differences between the control using *t*-test (^*^p < 0.05). **(B)** Shoot phenotype of 20-days old seedling which were grown on the agar medium containing the indicated compounds (Scale bar = 1 cm). cont. means control with only acetone at 0.1%.

As mentioned earlier, *cis*-CA, but not *trans*-CA, was reported to have plant growth-promoting activity at relatively low concentration by inhibiting the auxin efflux (Steenackers et al., 2017, 2019). Because *c*-CASL1 was found to be non-enzymatically degraded to some extent, we expected that *c-*CASL1 treatment might bring the same effect as treatment with *cis*-CA alone. To test this hypothesis, we grew *Arabidopsis* WT plants on the agar plates containing *c*-CASL1, *t*-CASL1, *cis*-CA, or *trans*-*CA. cis*-CA was reported to promote lateral root growth and thus to increase leaf size. We found that root growth phenotype was affected by not only *cis*-CA but also *c*-CASL1 (Supplementary Figure 7A). These two compounds almost equally inhibited the primary root length as well as increased the lateral root density (Supplementary Figure 7B). The root growth direction was also affected by *c*-CASL as was the case with *cis*-CA (Supplementary Figure 7A). Moreover, we observed an increase in shoot fresh weight after treatment with *cis*-CA or *c*-CASL (Figure 7). Thus, we conclude that *c*-CASL1 treatment produced the same effect as *cis*-CA treatment with growth promotion in *Arabidopsis*.

## Discussion

In this paper, we report successful development of a series of new SL analogs derived from CA, which is the basis of a class of phenylpropanoid compounds widely distributed in the plant kingdom. We synthesized 25 analogs, all of which showed moderate activity as suicidal germination inducers for the root parasitic plants, *O. minor* and *S. hermonthica.* The most active compounds, in which an electron withdrawing group was introduced at the C-4 position, showed almost equal activity to GR24. Interestingly, *cis*-CA-derived SL analogs showed stronger activity than the corresponding *trans* isomer-derived analogs. We also found that *cis*-CASLs interacted with the *S. hermonthica* receptor, HTL7 or HTL6, more strongly than the corresponding *trans*-CASLs. We also synthesized an analog from 3-PPA (denoted PPASL), in which the side-chain double bond was reduced to a single bond, and this analog showed very weak activity compared with the CA-derived analogs. However, *in vitro* YLG assay results showed that PPASL interacted directly with HTL7 with a relatively low IC_50_ value, as did other analogs. We still do not know the reason for the weak activity of this analog, but it might be possible that the uptake of this analog by the parasitic plant seeds is less effective than the CA derivatives, for some reason, or that this analog might be quickly metabolized *in planta*.

Because *cis*-CASL showed stronger activity inducing germination of the parasitic plants, we synthesized conformationally-fixed analogs by introducing a 5-member or 6-member ring structure. However, the activity of the conformationally-fixed *cis* isomer analogs (*c*-indCASL, or *c*-tetCASL) was not increased compared with the non-fixed analog, *c*-CASL1. In contrast, in the case of the *trans* isomer analogs, the fixed conformation analogs (*t*-indCASL, or *t*-tetCASL) showed much stronger activity compared with the non-fixed analog, *t*-CASL1. Therefore, introducing a fixed conformation decreased the activity difference between *cis* and *trans* isomers; however, this strategy would not be suitable as a method to obtain more potent analogs.

We also found that CA-derived SL analogs did not inhibit shoot branching in *Arabidopsis* when applied to the SL biosynthetic mutant, *max4*, at 1 μM. At a high concentration, 5 μM, some of these analogs slightly inhibited shoot branching. However, it was likely that the branching reduction was due to the growth inhibiting activity by a side effect of the tested compounds. If considering that these analogs were active as the germination inducers for root parasitic plants at concentrations well below 1 μM, these new analogs might provide new lead chemicals as suicidal germination inducers that do not affect the host plant architecture. Because *cis*-CA was reported to promote plant growth at relatively low concentration, we evaluated *c*-CASL1 for such activity. As a result, we found that not only *cis*-CA but also *c*-CASL1 showed growth-promoting activity toward the *Arabidopsis* WT plant. As CA-derived SLs were found to be degraded to some extent in the hydroponic culture medium, it is likely that the growth-promoting activity of *c*-CASL1 was simply a result of its degradation in the culture medium, leading to release of free *c*-CA. It would be also possible that CASLs are hydrolyzed to *cis*-CA in a manner dependent on the SL receptor, D14. In fact, CASLs were hydrolyzed by AtD14 or HTL7. Although we have not tested for activity toward other plants, including crop species, these new SL analogs might provide lead chemicals with two activities, as suicidal germination inducers for root parasitic plants as well as plant growth-promoting reagents for the host plants. This feature could be one of advantages compared with the previously reported SL analogs.

In conclusion, we successfully prepared new SL analogs derived from CA with useful biological activities. Many structural analogs of CA are commercially available, therefore it would be possible to prepare more diverse analogs to obtain further potent agonists.

## Data Availability Statement

The original contributions presented in the study are included in the article/Supplementary Material; further inquiries can be directed to the corresponding author.

## Author Contributions

TS performed the majority of the experiments. MK performed a part of the YLG assay experiment. YS designed the research. TS, MK, and YS wrote the manuscript. All authors contributed to the article and approved the submitted version.

## Funding

This work was supported by MEXT KAKENHI grant number 19K05852, Mitsubishi Foundation, and Kato Memorial Bioscience Foundation.

## Conflict of Interest

The authors declare that they have no known competing financial interests or personal relationships that could have appeared to influence the work reported in this paper.

## Publisher’s Note

All claims expressed in this article are solely those of the authors and do not necessarily represent those of their affiliated organizations, or those of the publisher, the editors and the reviewers. Any product that may be evaluated in this article, or claim that may be made by its manufacturer, is not guaranteed or endorsed by the publisher.

## References

[ref1] Akiyama K. Matsuzaki K. Hayashi H. (2005). Plant sesquiterpenes induce hyphal branching in arbuscular mycorrhizal fungi. Nature 435, 824–827. doi: 10.1038/nature03608, PMID: 15944706

[ref2] Arite T. Umehara M. Ishikawa S. Hanada A. Maekawa M. Yamaguchi S.. (2009). d14, a strigolactone-insensitive mutant of rice, shows an accelerated outgrowth of tillers. Plant Cell Physiol. 50, 1416–1424. doi: 10.1093/pcp/pcp091, PMID: 19542179

[ref3] Conn C. E. Bythell-Douglas R. Neumann D. Yoshida S. Whittington B. Westwood J. H.. (2015). Plant evolution. Convergent evolution of strigolactone perception enabled host detection in parasitic plants. Science 349, 540–543. doi: 10.1126/science.aab1140, PMID: 26228149

[ref4] Cook C. E. Whichard L. P. Turner B. Wall M. E. Egley G. H. (1966). Germination of Witchweed (Striga lutea Lour.): isolation and properties of a potent stimulant. Science 154, 1189–1190. doi: 10.1126/science.154.3753.1189, PMID: 17780042

[ref5] de Saint Germain A. Clave G. Badet-Denisot M. A. Pillot J. P. Cornu D. Le Caer J. P.. (2016). An histidine covalent receptor and butenolide complex mediates strigolactone perception. Nat. Chem. Biol. 12, 787–794. doi: 10.1038/nchembio.2147, PMID: 27479744 PMC5030144

[ref6] Fukui K. Ito S. Ueno K. Yamaguchi S. Kyozuka J. Asami T. (2011). New branching inhibitors and their potential as strigolactone mimics in rice. Bioorg. Med. Chem. Lett. 21, 4905–4908. doi: 10.1016/j.bmcl.2011.06.019, PMID: 21741836

[ref7] Gomez-Roldan V. Fermas S. Brewer P. B. Puech-Pages V. Dun E. A. Pillot J. P.. (2008). Strigolactone inhibition of shoot branching. Nature 455, 189–194. doi: 10.1038/nature07271 18690209

[ref8] Hamiaux C. Drummond R. S. Janssen B. J. Ledger S. E. Cooney J. M. Newcomb R. D.. (2012). DAD2 is an alpha/beta hydrolase likely to be involved in the perception of the plant branching hormone, strigolactone. Curr. Biol. 22, 2032–2036. doi: 10.1016/j.cub.2012.08.007, PMID: 22959345

[ref9] Hiradate S. Morita S. Furubayashi A. Fujii Y. Harada J. (2005). Plant growth inhibition by cis-cinnamoyl glucosides and cis-cinnamic acid. J. Chem. Ecol. 31, 591–601. doi: 10.1007/s10886-005-2047-0, PMID: 15898503

[ref10] Mashiguchi K. Seto Y. Yamaguchi S. (2021). Strigolactone biosynthesis, transport and perception. Plant J. 105, 335–350. doi: 10.1111/tpj.15059 33118266

[ref11] Nishikawa K. Fukuda H. Abe M. Nakanishi K. Tazawa Y. Yamaguchi C.. (2013). Design and synthesis of conformationally constrained analogues of cis-cinnamic acid and evaluation of their plant growth inhibitory activity. Phytochemistry 96, 223–234. doi: 10.1016/j.phytochem.2013.10.001, PMID: 24176527

[ref001] Noren H. Svensson P. Andersson B. (2004). A convenient and versatile hydroponic cultivation system for *Arabidopsis thaliana*. Physiol. Plant. 121, 343–348. doi: 10.1111/j.0031-9317.2004.00350.x

[ref12] Seto Y. Yasui R. Kameoka H. Tamiru M. Cao M. Terauchi R.. (2019). Strigolactone perception and deactivation by a hydrolase receptor DWARF14. Nat. Commun. 10:191. doi: 10.1038/s41467-018-08124-7, PMID: 30643123 PMC6331613

[ref13] Steenackers W. El Houari I. Baekelandt A. Witvrouw K. Dhondt S. Leroux O.. (2019). cis-Cinnamic acid is a natural plant growth-promoting compound. J. Exp. Bot. 70, 6293–6304. doi: 10.1093/jxb/erz392, PMID: 31504728 PMC6859716

[ref14] Steenackers W. Klima P. Quareshy M. Cesarino I. Kumpf R. P. Corneillie S.. (2017). cis-Cinnamic acid is a novel, natural auxin efflux inhibitor that promotes lateral root formation. Plant Physiol. 173, 552–565. doi: 10.1104/pp.16.00943, PMID: 27837086 PMC5210711

[ref15] Toh S. Holbrook-Smith D. Stogios P. J. Onopriyenko O. Lumba S. Tsuchiya Y.. (2015). Structure-function analysis identifies highly sensitive strigolactone receptors in Striga. Science 350, 203–207. doi: 10.1126/science.aac9476, PMID: 26450211

[ref16] Tsuchiya Y. Yoshimura M. Hagihara S. (2018). The dynamics of strigolactone perception in *Striga hermonthica*: a working hypothesis. J. Exp. Bot. 69, 2281–2290. doi: 10.1093/jxb/ery061, PMID: 29474634

[ref17] Tsuchiya Y. Yoshimura M. Sato Y. Kuwata K. Toh S. Holbrook-Smith D.. (2015). Parasitic plants. Probing strigolactone receptors in *Striga hermonthica* with fluorescence. Science 349, 864–868. doi: 10.1126/science.aab3831, PMID: 26293962

[ref18] Umehara M. Cao M. Akiyama K. Akatsu T. Seto Y. Hanada A.. (2015). Structural requirements of strigolactones for shoot branching inhibition in rice and *Arabidopsis*. Plant Cell Physiol. 56, 1059–1072. doi: 10.1093/pcp/pcv028, PMID: 25713176

[ref19] Umehara M. Hanada A. Yoshida S. Akiyama K. Arite T. Takeda-Kamiya N.. (2008). Inhibition of shoot branching by new terpenoid plant hormones. Nature 455, 195–200. doi: 10.1038/nature07272, PMID: 18690207

[ref20] Uraguchi D. Kuwata K. Hijikata Y. Yamaguchi R. Imaizumi H. Am S.. (2018). A femtomolar-range suicide germination stimulant for the parasitic plant *Striga hermonthica*. Science 362, 1301–1305. doi: 10.1126/science.aau5445, PMID: 30545887

[ref21] Waters M. T. Nelson D. C. Scaffidi A. Flematti G. R. Sun Y. K. Dixon K. W.. (2012). Specialisation within the DWARF14 protein family confers distinct responses to karrikins and strigolactones in *Arabidopsis*. Development 139, 1285–1295. doi: 10.1242/dev.074567, PMID: 22357928

[ref22] Yang X. X. Choi H. W. Yang S. F. Li N. (1999). A UV-light activated cinnamic acid isomer regulates plant growth and gravitropism via an ethylene receptor-independent pathway. Aust. J. Plant Physiol. 26, 325–335. doi: 10.1071/PP99007, PMID: 11542914

[ref23] Yao R. Ming Z. Yan L. Li S. Wang F. Ma S.. (2016). DWARF14 is a non-canonical hormone receptor for strigolactone. Nature 536, 469–473. doi: 10.1038/nature19073, PMID: 27479325

