# Supplementary material for: A New Series of Strigolactone Analogs Derived From Cinnamic Acids as Germination Inducers for Root Parasitic Plants
Source: Front Plant Sci. 2022 Mar 29;13:843362. doi: 10.3389/fpls.2022.843362 (PMC9002265; doi:10.3389/fpls.2022.843362)
Supplement: Supplementary file 1 [file Data_Sheet_1.PDF]

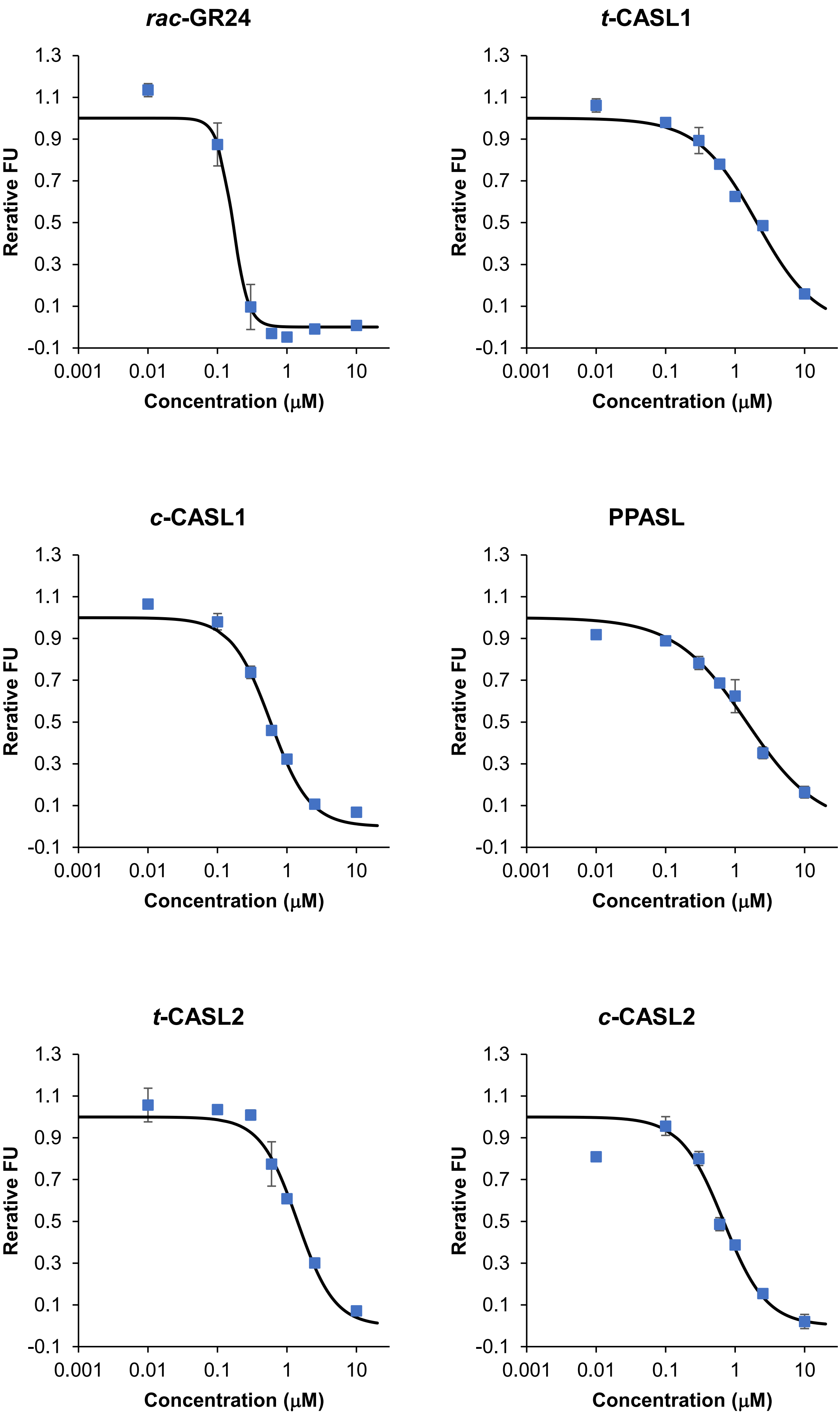

rac-GR24
t-CASL1
c-CASL1
PPASL
t-CASL2
c-CASL2
Rerative FU
1.3
1.1
0.9
0.7
0.5
0.3
0.1
-0.1
0.001
0.01
0.1
1
10
Concentration (μM)

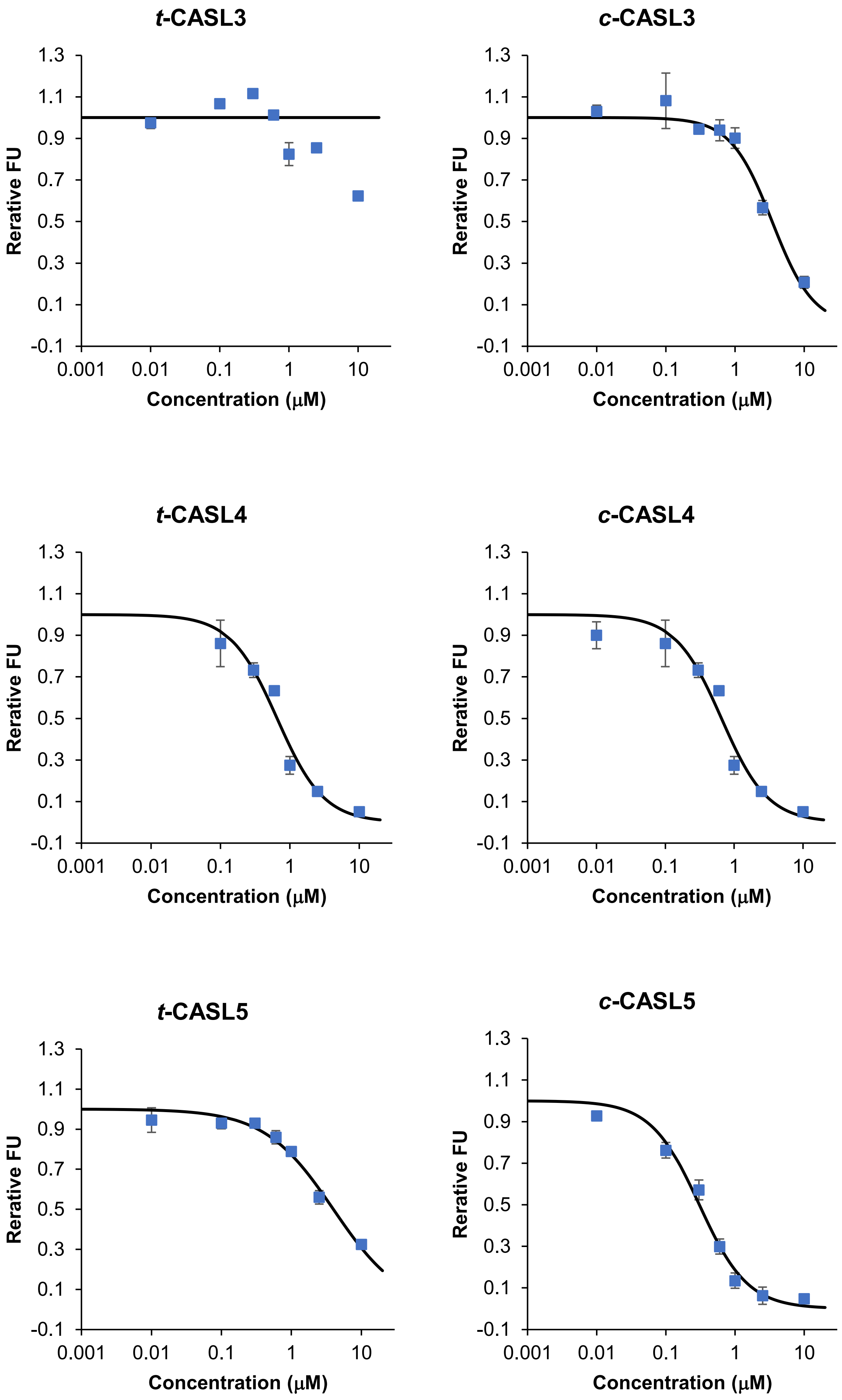
t-CASL3
c-CASL3
t-CASL4
c-CASL4
t-CASL5
c-CASL5
Rerative FU
Concentration (μM)
1.3
1.1
0.9
0.7
0.5
0.3
0.1
-0.1
0.001
0.01
0.1
1
10

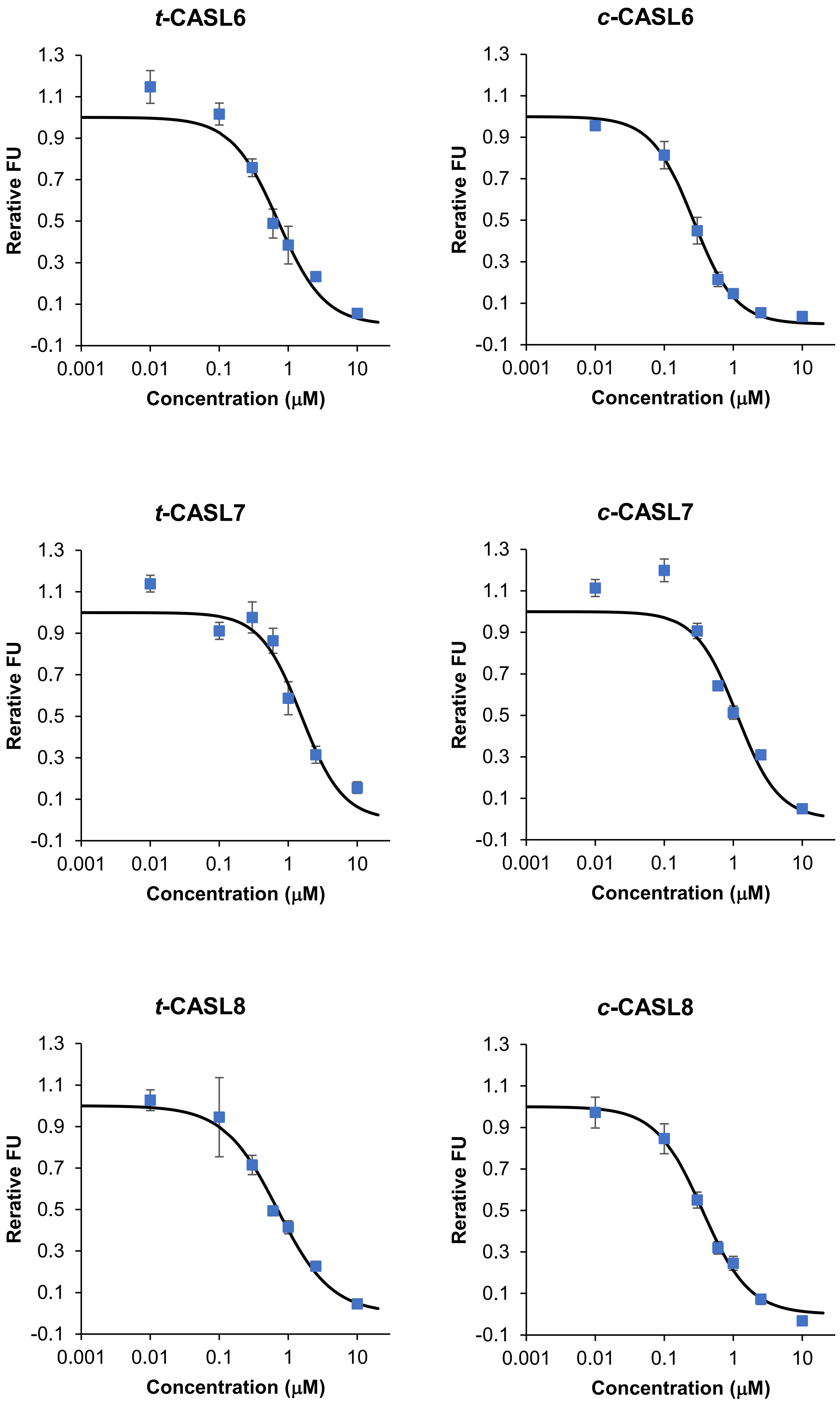
t-CASL6
c-CASL6
t-CASL7
c-CASL7
t-CASL8
c-CASL8
Rerative FU
Concentration (μM)
1.3
1.1
0.9
0.7
0.5
0.3
0.1
-0.1
0.001
0.01
0.1
1
10

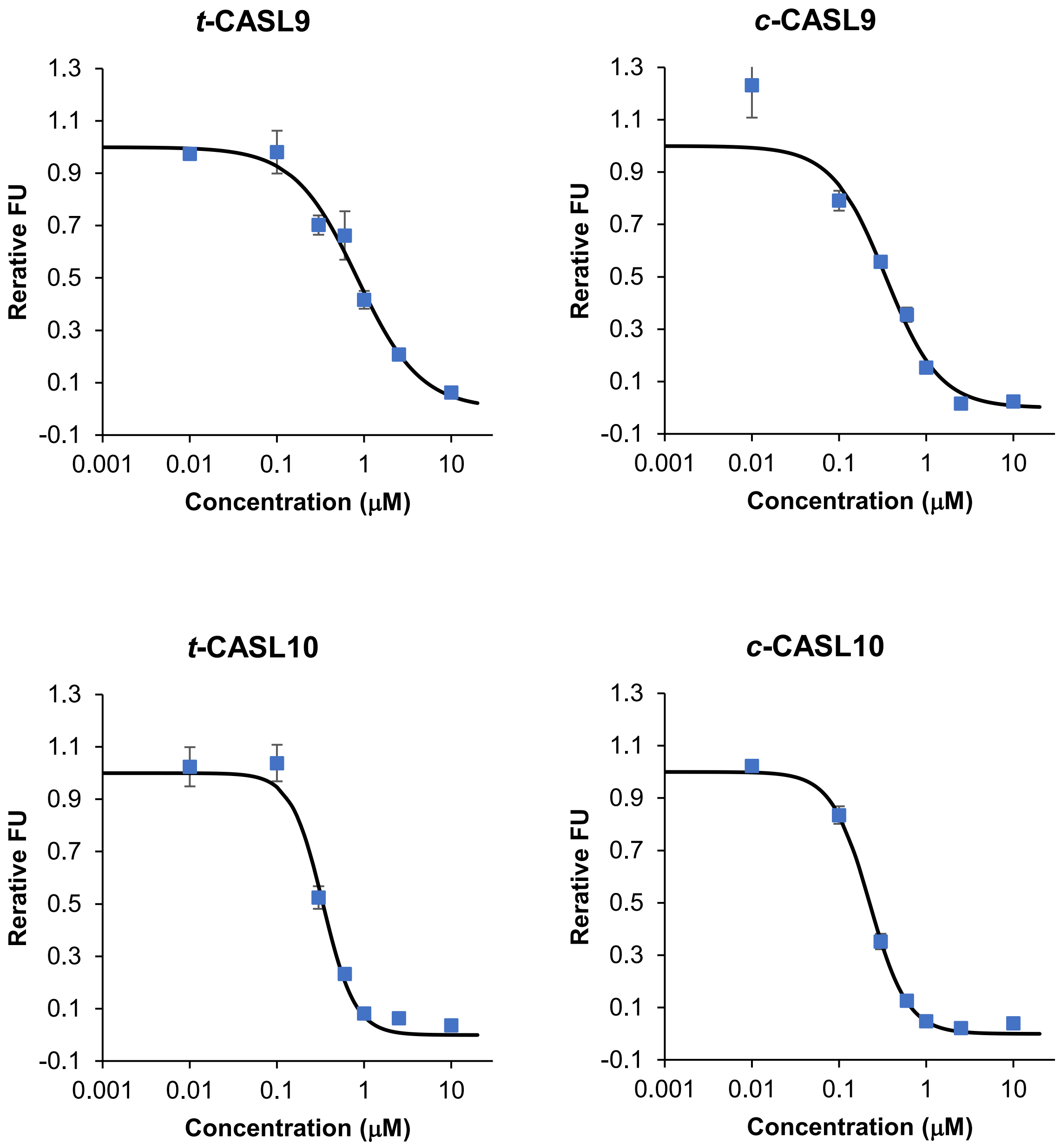

t-CASL9
c-CASL9
t-CASL10
c-CASL10
Rerative FU
Concentration (μM)
1.3
1.1
0.9
0.7
0.5
0.3
0.1
-0.1
0.001
0.01
0.1
1
10

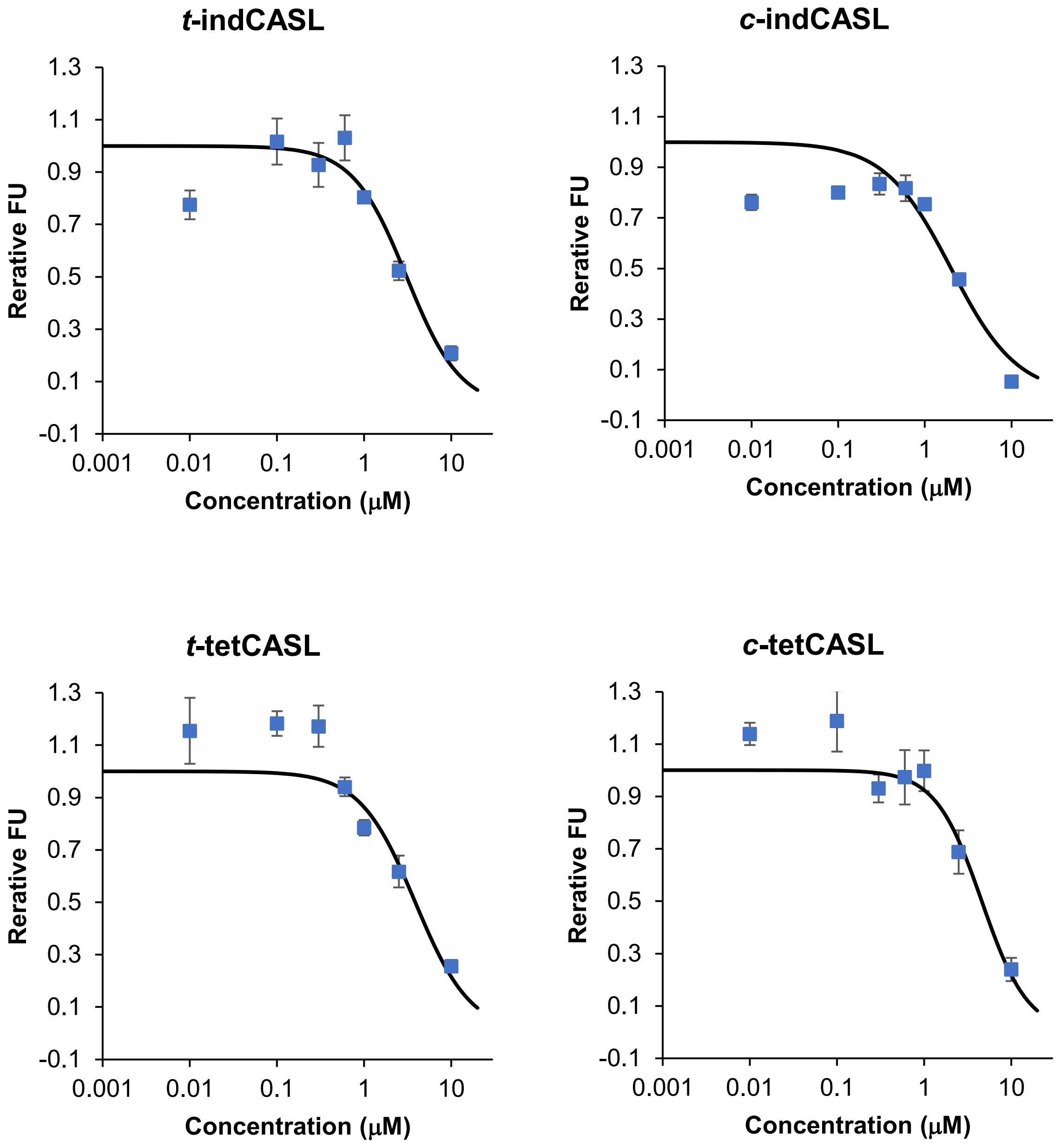

Supplementary figure1 | The results of YLG assay using each CASL and ShHTL7. Data are the means ± SD (n=3). The graphs were made by using the online tool Quest GraphTM IC50 Calculator (AAT Bioquest, Inc., USA).

Compounds	IC_{50} (μM)
rac-GR24	2.35
t-CASL1	1.85
c-CASL1	0.90
PPASL	2.24

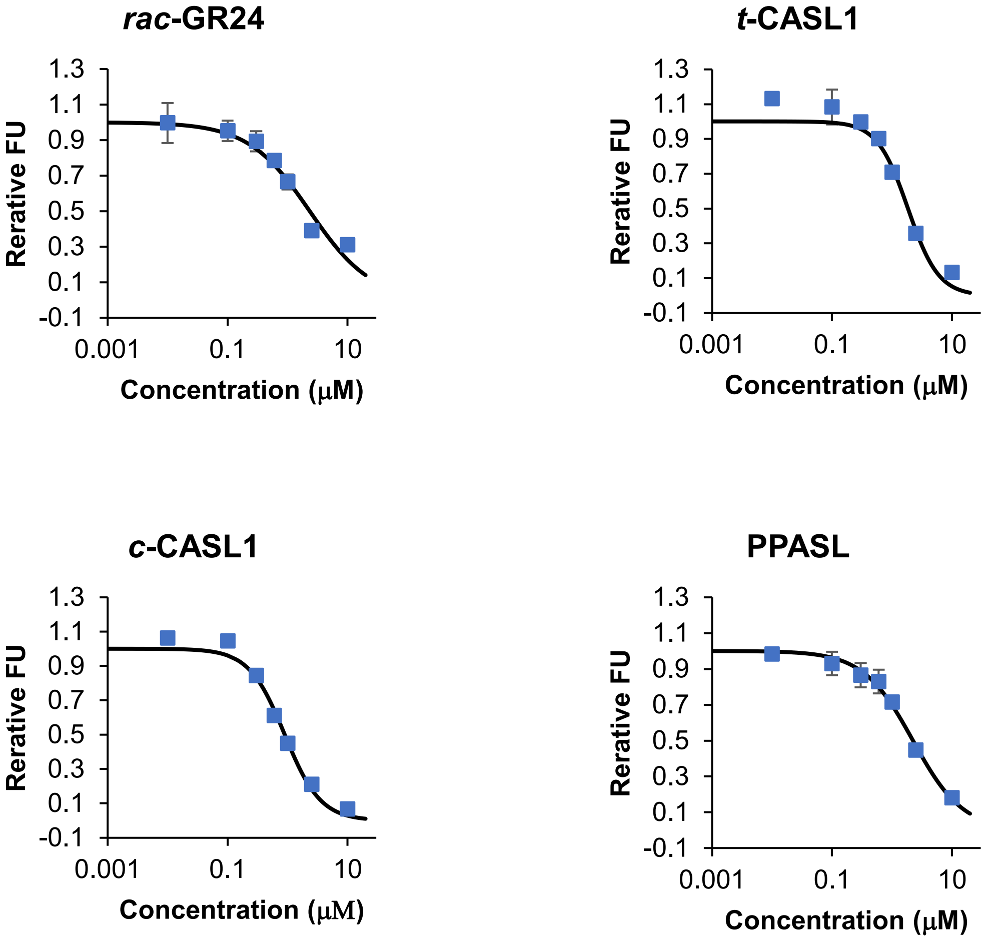

Supplementary figure 2 | The results of YLG assay using each CASL and ShHTL6. Data are the means ± SD (n=3). The graphs were made by using the online tool Quest GraphTM IC50 Calculator (AAT Bioquest, Inc., USA).

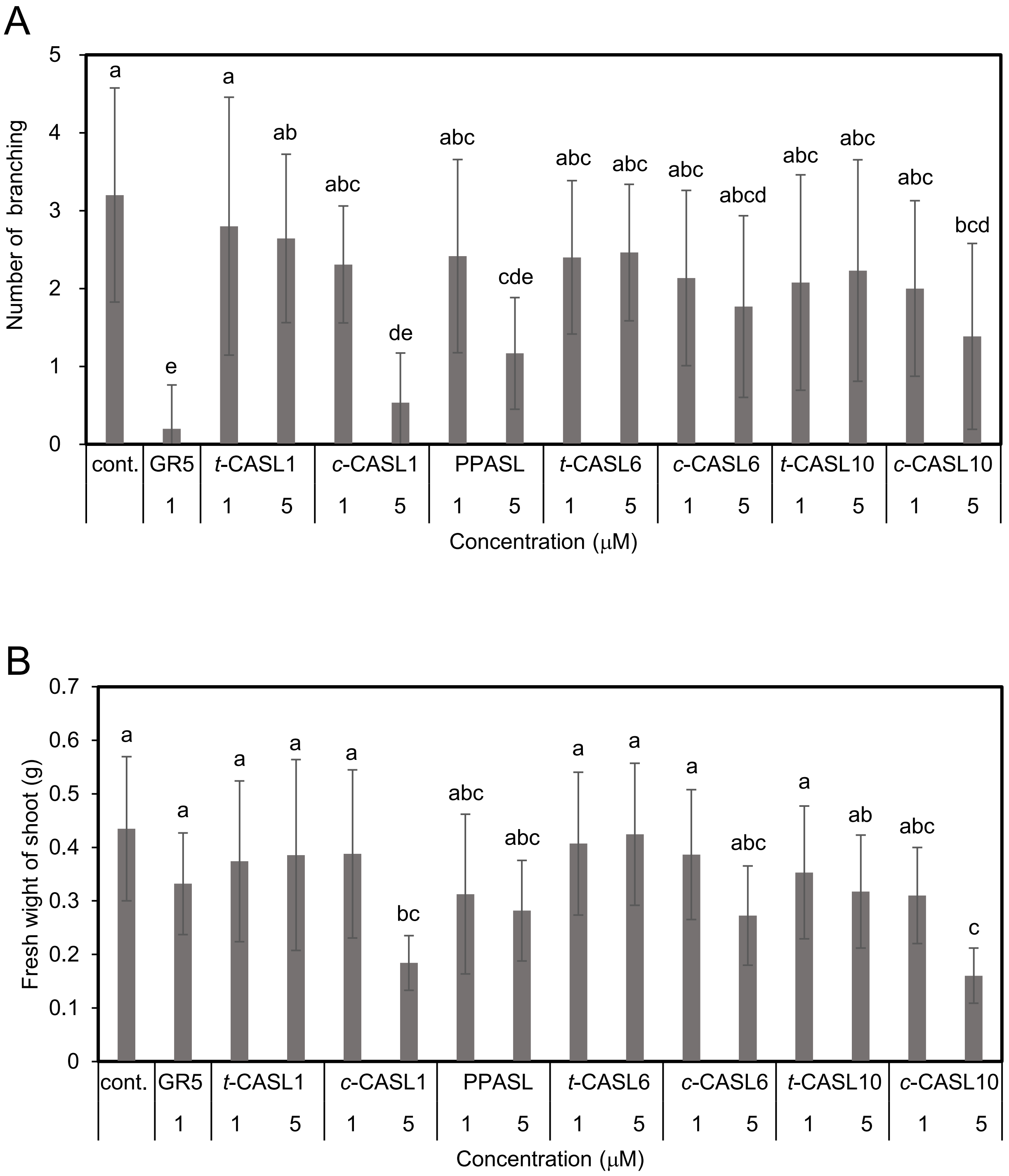

A
Number of branching
0
1
2
3
4
5
a
e
a
ab
abc
de
abc
cde
abc
abc
abc
abcd
abc
abc
abc
bcd
cont.
GR5
t-CASL1
c-CASL1
PPASL
t-CASL6
c-CASL6
t-CASL10
c-CASL10
1 1 5 1 5 1 5 1 5 1 5 1 5 1 5
Concentration (μM)
B
Fresh wight of shoot (g)
0
0.1
0.2
0.3
0.4
0.5
0.6
0.7
a
a
a
a
a
bc
abc
abc
a
a
a
abc
a
ab
abc
c
cont.
GR5
t-CASL1
c-CASL1
PPASL
t-CASL6
c-CASL6
t-CASL10
c-CASL10
1 1 5 1 5 1 5 1 5 1 5 1 5 1 5
Concentration (μM)

C

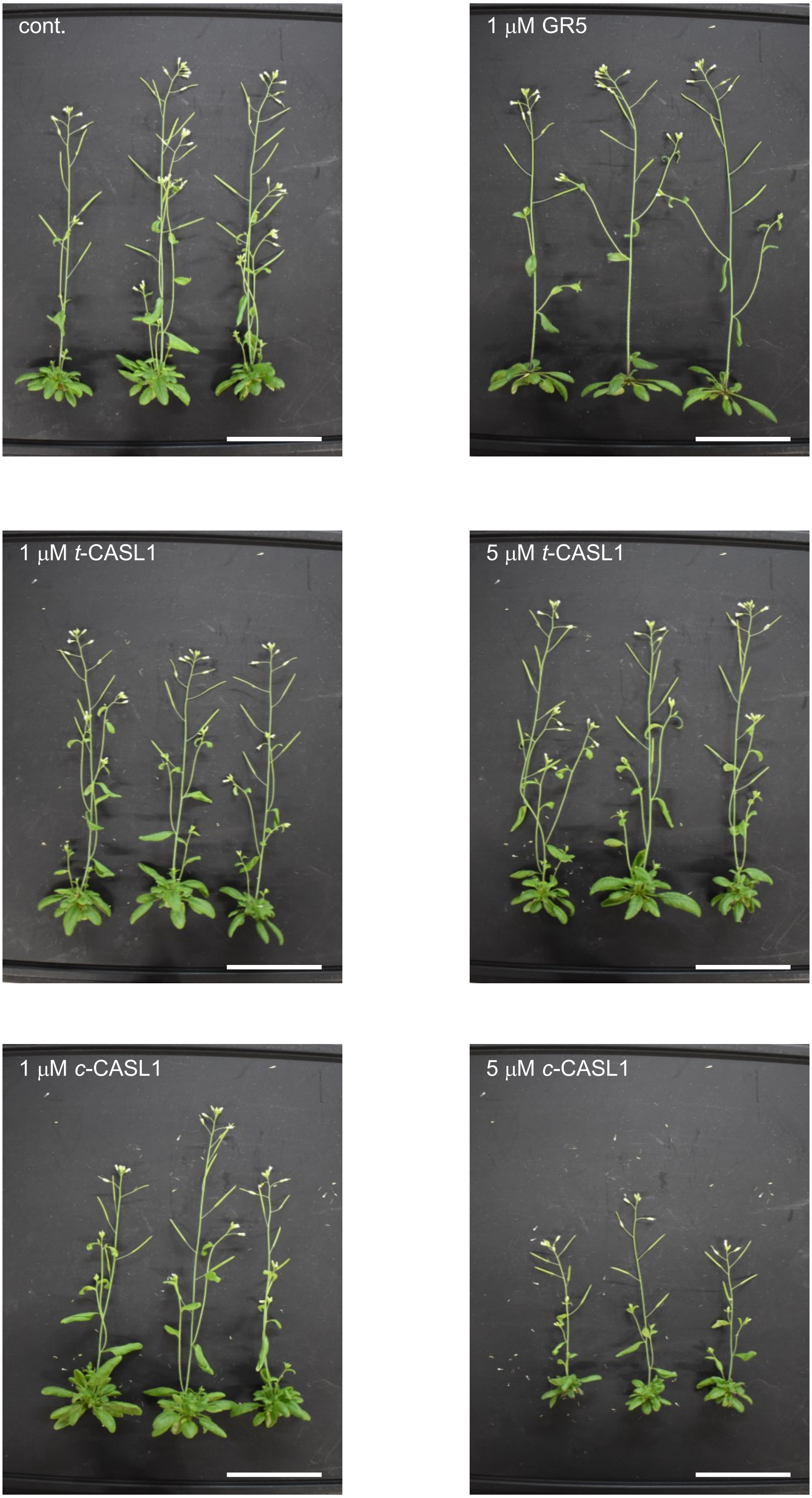

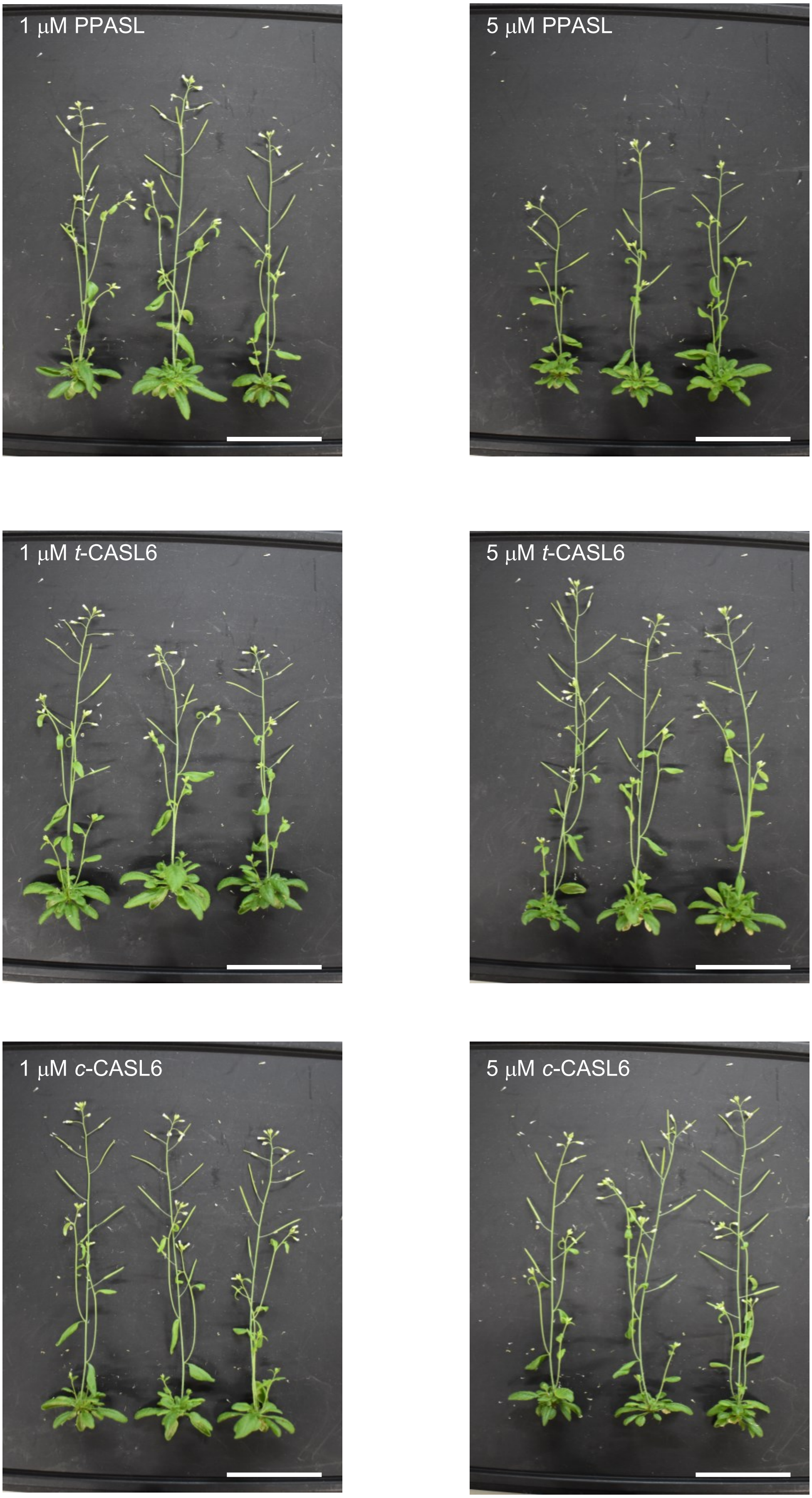
1 μM PPASL
5 μM PPASL
1 μM t-CASL6
5 μM t-CASL6
1 μM c-CASL6
5 μM c-CASL6

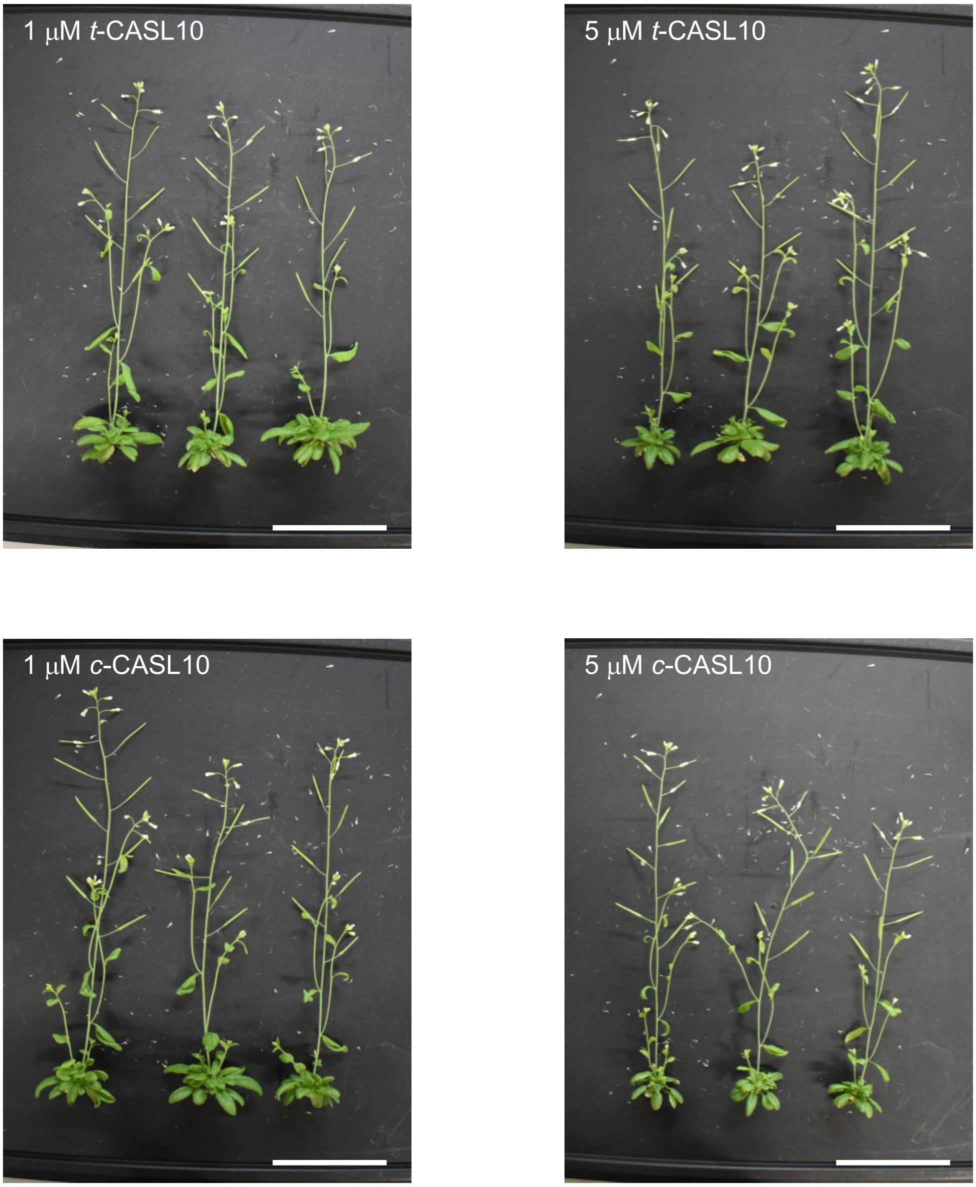

Supplementary figure.3 | Shoot branching inhibiting activity of CASLs at 5 μM using the Arabidopsis *max4* mutant. (A) Number of axillary shoots (over 5 mm) of *max4* which were grown in hydroponic culture containing test compounds. Data are the means ± SD (n=12-15). Different letters indicate significant differences at P <0.05 with Tukey multiple comparison test. (B) Shoot fresh weight of Arabidopsis plants after treatment of each CASL or PPASL. Data are the means ± SD (n=12-15). Different letters indicate significant differences at P <0.05 with Tukey multiple comparison test. (C) Shoot phenotype of Arabidopsis plants after treatment of each CASL or PPASL (Scale bar=5 cm). Cont. means control with only acetone at 0.01%.

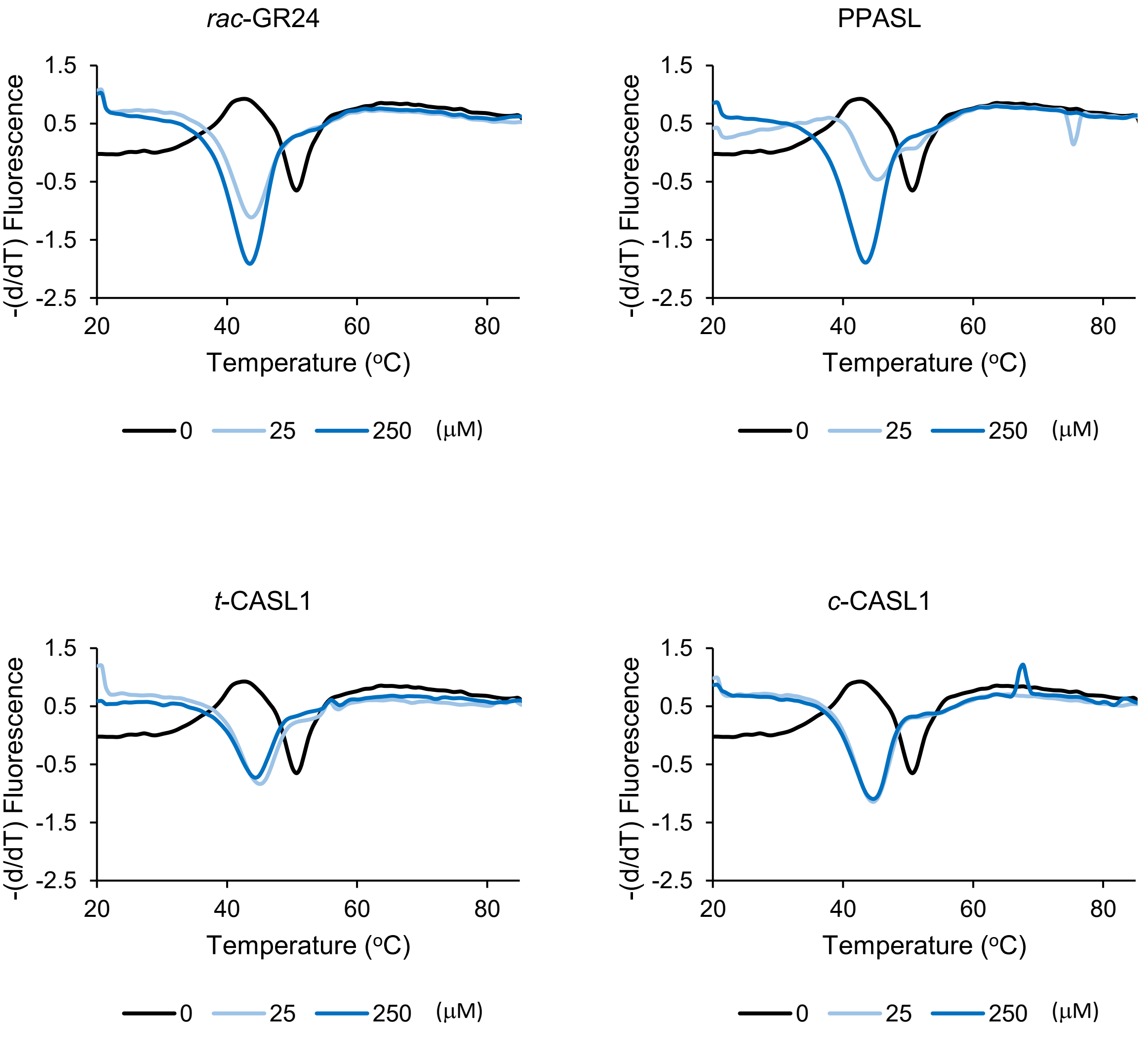

Supplementary figure.4 | DSF analysis of AtD14 in the presence of each CASL or PPASL. Melting temperature curves of AtD14 which was incubated with indicated concentration of each chemical are shown. Data are the means (n=3).

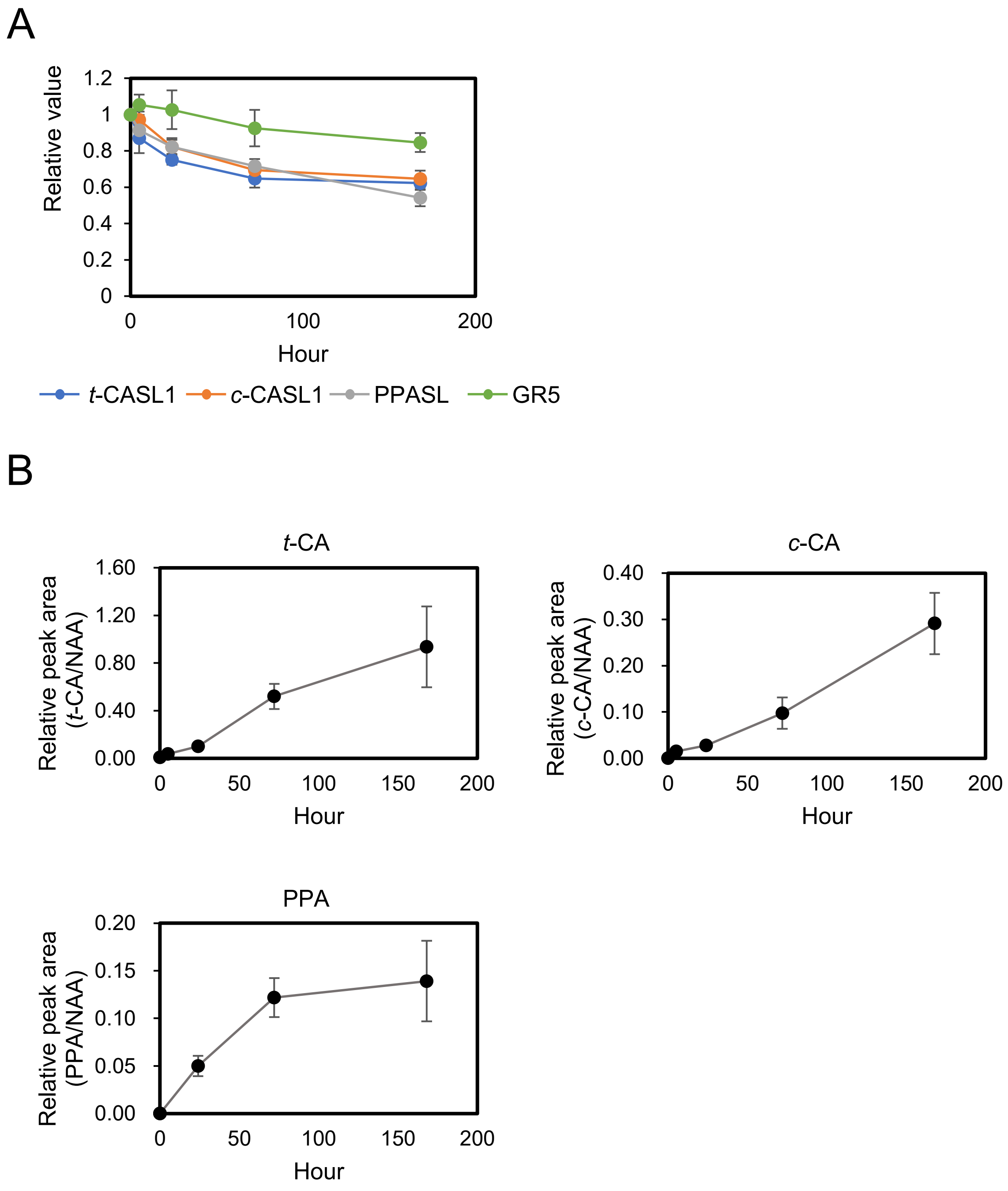

Supplementary figure 5 | Time course analysis of CASLs and PPASL stability in the hydroponic culture (pH 5.7). (A) Relative amount of test compounds calculated by using LC-MS/MS analysis. Data are the means ± SD (n=3). (B) Relative peak area of the released CAs or PPA compared with the internal standard (NAA). Data are the means ± SD (n=3).

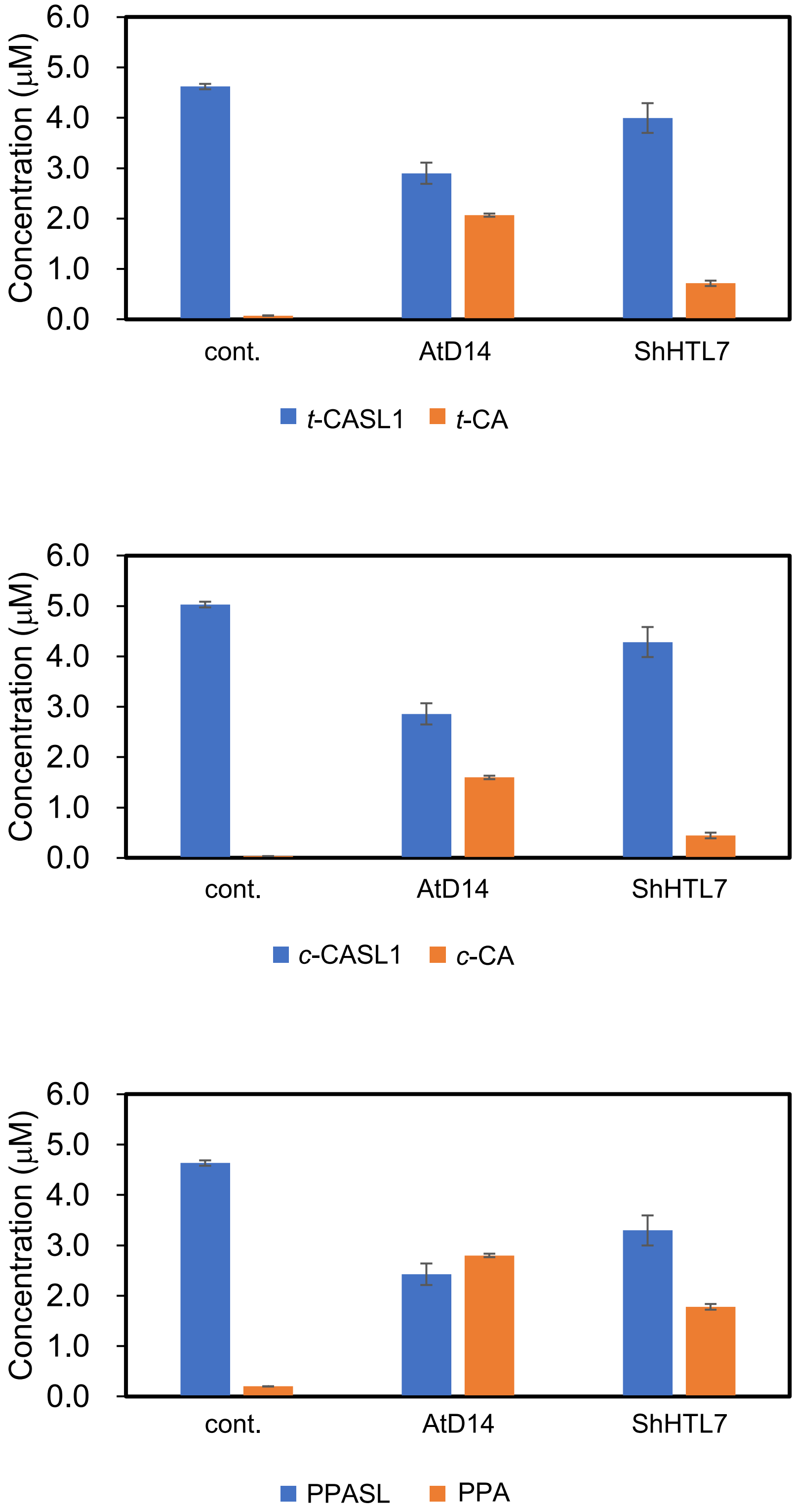

Supplementary figure 6 | Enzymatic degradation of CASLs and PPASL by AtD14 or ShHTL7 after 60 min incubation. Concentration of the remaining substrate and the product was calculated by using LC-MS/MS. Data are the means ± SD (n=3). Cont. means the reaction with no protein.

A

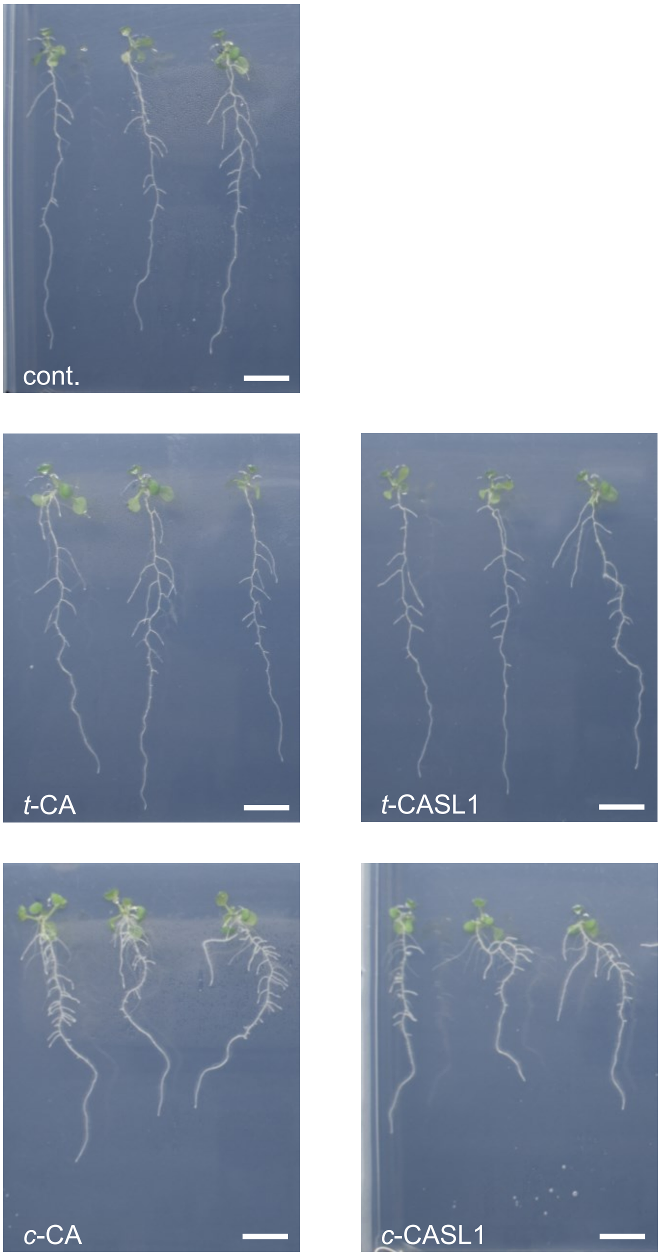

B

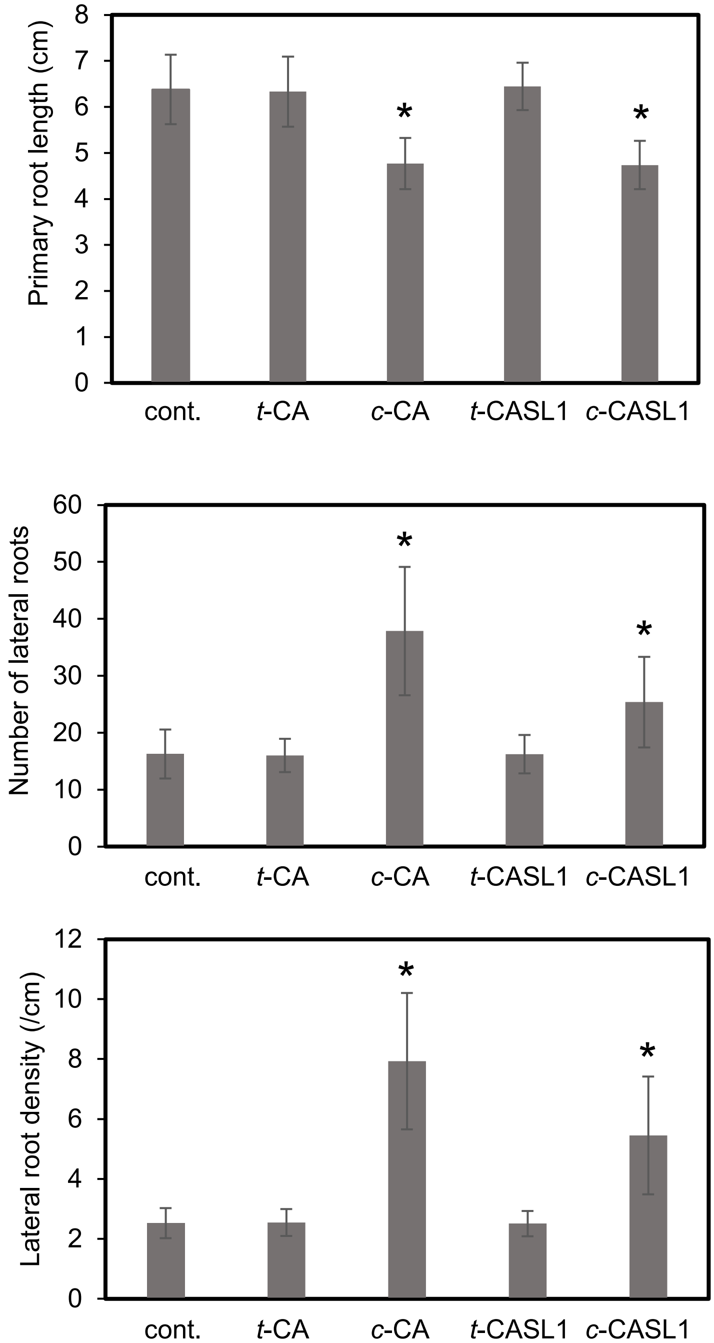

Supplementary figure 7 | Root phenotype analysis of CA or CASL treated plants. (A) Root morphological phenotype of the Arabidopsis WT Col-0 seedlings after treatment of each CA or CASL at 1 μM concentration for 11 days (scale bar=1 cm). (B) Primary root length (top panel), number of lateral roots (middle panel) and lateral root density (bottom panel) of after 11 days cultivation. Data are the means ± SD (n=13-14). Asterisks indicate the significant differences between the control using *t*-test (*$P < 0.05$). Cont. means control in the absence of protein.

Supplementary table 1 | The information of LC-MS/MS analytical condition.

Compounds	MW	Parent ion (*m/z*)	Used fragment ion (*m/z*)	Experiment
t-CASL1	244	245	97.03	Hydrolysis assay
t-CA	148	149	131.05	Hydrolysis assay
c-CASL1	244	245	97.03	Hydrolysis assay
c-CA	148	149	131.05	Hydrolysis assay
PPASL	246	247	97.03	Hydrolysis assay
PPA	150	151	105.07	Hydrolysis assay
NAA	186	187	141.07	Hydrolysis assay
t-CASL1	244	245	131.05	Time course monitoring
t-CA	148	149	131.05	Time course monitoring
c-CASL1	244	245	131.05	Time course monitoring
c-CA	148	149	131.05	Time course monitoring
PPASL	246	247	133.06	Time course monitoring
PPA	150	151	105.07	Time course monitoring
NAA	186	187	141.07	Time course monitoring
GR5	210	211	97.03	Time course monitoring
